# Evidence-based potential of generative artificial intelligence large language models in orthodontics: a comparative study of ChatGPT, Google Bard, and Microsoft Bing

**DOI:** 10.1093/ejo/cjae017

**Published:** 2024-04-13

**Authors:** Miltiadis A Makrygiannakis, Kostis Giannakopoulos, Eleftherios G Kaklamanos

**Affiliations:** School of Dentistry, National and Kapodistrian University of Athens, Athens 11527, Greece; School of Dentistry, European University Cyprus, Nicosia 2404, Cyprus; School of Dentistry, European University Cyprus, Nicosia 2404, Cyprus; School of Dentistry, European University Cyprus, Nicosia 2404, Cyprus; School of Dentistry, Aristotle University of Thessaloniki, Thessaloniki 54124, Greece; Hamdan bin Mohammed College of Dental Medicine, Mohammed bin Rashid University of Medicine and Health Sciences (MBRU), Dubai 505055, United Arab Emirates

**Keywords:** orthodontics, large language models, ChatGPT, Google bard, Microsoft bing chat

## Abstract

**Background:**

The increasing utilization of large language models (LLMs) in Generative Artificial Intelligence across various medical and dental fields, and specifically orthodontics, raises questions about their accuracy.

**Objective:**

This study aimed to assess and compare the answers offered by four LLMs: Google’s Bard, OpenAI’s ChatGPT-3.5, and ChatGPT-4, and Microsoft’s Bing, in response to clinically relevant questions within the field of orthodontics.

**Materials and methods:**

Ten open-type clinical orthodontics-related questions were posed to the LLMs. The responses provided by the LLMs were assessed on a scale ranging from 0 (minimum) to 10 (maximum) points, benchmarked against robust scientific evidence, including consensus statements and systematic reviews, using a predefined rubric. After a 4-week interval from the initial evaluation, the answers were reevaluated to gauge intra-evaluator reliability. Statistical comparisons were conducted on the scores using Friedman’s and Wilcoxon’s tests to identify the model providing the answers with the most comprehensiveness, scientific accuracy, clarity, and relevance.

**Results:**

Overall, no statistically significant differences between the scores given by the two evaluators, on both scoring occasions, were detected, so an average score for every LLM was computed. The LLM answers scoring the highest, were those of Microsoft Bing Chat (average score = 7.1), followed by ChatGPT 4 (average score = 4.7), Google Bard (average score = 4.6), and finally ChatGPT 3.5 (average score 3.8). While Microsoft Bing Chat statistically outperformed ChatGPT-3.5 (*P*-value = 0.017) and Google Bard (*P*-value = 0.029), as well, and Chat GPT-4 outperformed Chat GPT-3.5 (*P*-value = 0.011), all models occasionally produced answers with a lack of comprehensiveness, scientific accuracy, clarity, and relevance.

**Limitations:**

The questions asked were indicative and did not cover the entire field of orthodontics.

**Conclusions:**

Language models (LLMs) show great potential in supporting evidence-based orthodontics. However, their current limitations pose a potential risk of making incorrect healthcare decisions if utilized without careful consideration. Consequently, these tools cannot serve as a substitute for the orthodontist’s essential critical thinking and comprehensive subject knowledge. For effective integration into practice, further research, clinical validation, and enhancements to the models are essential. Clinicians must be mindful of the limitations of LLMs, as their imprudent utilization could have adverse effects on patient care.

## Introduction

By the end of 2022, a groundbreaking advance in artificial intelligence (AI) technology was unveiled: ChatGPT by OpenAI Inc. (San Francisco, CA, USA). In the very first 3 months following its inauguration, it already had an astonishing 100 million new users [1]. Over the past few years, there has been a remarkable growth in AI applications and tools in the area of dentistry, as well. The primary objective of the implementation of AI in this discipline is helping professionals in offering improved oral healthcare services. According to a recently published white paper, these tools are capable of supporting a number of functions, such as image analysis, radiograph interpretation, use of neural networks for diagnoses, data synthesis, information on materials, and clinical techniques to improve outcomes, management of patient records, applications in forensic dentistry, orthodontics, periodontology, endodontics, caries diagnosis, treatment planning, and even facilitation in communication and interaction with patients [2]. With the integration of AI technology, clinical queries can be readily addressed on a mobile phone, and ongoing education updates can be easily delivered [2–8]. When considerately combined with a dentist’s clinical expertise and a patient’s treatment needs and preferences, AI may help busy clinicians confront and overcome challenges associated with applying evidence-based dentistry [9–11]. In this way, AI and especially Generative AI (GenAI), which is a form of AI model capable of taking raw data and “learn” to generate statistically probable outputs (including text, images, audio, video, software code) when prompted [12], could potentially help clinicians provide customized, patient-centered care, and reinforce a more efficient and reliable clinical practice [13].

ChatGPT, specifically, which is categorized as a Large Language Model (LLM), is rooted in natural language processing (NLP), a facet of AI focused on enabling computers to comprehend natural language inputs. This involves utilizing various techniques such as machine learning and NLP [1, 14]. LLMs, in general, are neural networks extensively trained on vast text datasets from the Internet (comprising Wikipedia, digitized books, articles, and webpages). Their purpose is to process and generate coherent, human-like conversational responses based on the contextual input text (question or prompt). This is accomplished through deep-learning algorithms and advanced modeling [14–16]. Modern LLMs employ neural architecture based on positional encoding and self-attention techniques, enabling them to discern relationships within the input text and generate meaningful and relevant responses [16]. They exhibit the capacity to address follow-up questions, seek clarifications, challenge inaccuracies, and reject inappropriate requests [16]. Additionally, LLMs can be fine-tuned using reinforcement learning from human feedback to enhance their performance on specific tasks or specialized applications. This iterative process enhances their usability, accuracy, and functionality [17, 18].

Nowadays, various LLMs have emerged. The above-mentioned freely accessible version of ChatGPT is based on the GPT-3.5 language model, while the newer GPT-4 version is available exclusively under the ChatGPT Plus paid subscription. Subsequently, in February 2023, Microsoft (Microsoft Corporation, Redmond, WA, USA) introduced the Bing Chat AI chatbot utilizing the GPT-4 language model. In March 2023, Google (Google Ireland Limited, Dublin, Ireland) launched the Bard chatbot, initially powered by LaMDA (its proprietary family of LLMs) and later by PaLM 2 LLM. Among them, ChatGPT-3.5 and its enhanced subscription counterpart, ChatGPT-4, stand out in terms of user-friendliness and accessibility, being readily available to all on OpenAI’s website. This broad accessibility positions these bots as the preferred choice for many users. Meanwhile, Bing Chat, while boasting strengths such as suitability for research, live internet access, and compatibility with GPT-4, faces a notable limitation in its accessibility. With a chat limit of 100 requests per day, compared to ChatGPT’s allowance of 70 requests per hour, Bing Chat can potentially act as a bottleneck in a research study. This, combined with its restricted browser compatibility, renders it suboptimal for everyday use. On the other hand, Google Bard, now Gemini, despite having live internet access, is, at the time of writing, still in its early technological and commercial stages [19, 20].

The objective of the present study is to explore the current evidence-based potential of Generative Artificial Intelligence LLMs in orthodontics by comparatively assessing the answers provided by four LLMs: Google’s Bard, OpenAI’s ChatGPT-3.5, and ChatGPT-4, and Microsoft’s Bing, in response to clinically relevant questions within the field of orthodontics.

## Materials and methods

Ten indicative questions relevant to common clinical issues in orthodontics were asked of four different LLMs (Supplementary Table 1). The LLMs tested were: (i) ChatGPT model GPT-3.5 (offered for free at the moment). (ii) ChatGPT model GPT-4 (offered at ChatGTP Plus under subscription). (iii) Google Bard. (iv) Microsoft Bing search engine—chat function.

The questions used were agreed upon among the authors and had evidence to support the answers from consensus statements issued by scientific organizations or professional bodies, as well as from medical libraries and a PubMed database search for systematic reviews in high-impact factor, peer-reviewed scientific journals. All evidence retrieved served as the ‘gold standard’ with which the LLMs’ responses were compared.

Questions/prompts were written using appropriate terminology, and they were open-ended questions requiring a text-based response. Each question was asked once to each LLM by one of the authors, with no follow-up questions, rephrasing, or additional explanation in case of the LLM’s inability to answer. It was also not asked a second time by another author. By simulating scenarios where oral healthcare professionals seek immediate assistance with single questions, our study mirrored real-world situations. This approach made it easier to assess, under, to the extent possible, controlled conditions, how the LLMs could assist orthodontists in quick, on-demand information retrieval and clarification—a valuable skill in healthcare practice. Moreover, limiting interactions to single queries allowed for a more focused evaluation of the LLMs’ ability to provide concise and relevant responses to complex queries, without the need of re-prompting, meaning that the process can be one-off and not time-consuming.

Two evaluators assessed independently every answer from the four LLMs to each question. Both were specialist orthodontists practicing exclusively orthodontics and involved in undergraduate and postgraduate teaching of orthodontics. One of them is a PhD holder and a university faculty member, while the other one is a PhD candidate. The answer to each question was evaluated and graded in a range from 0 (minimum) to 10 (maximum) points against a rubric (Supplementary Table 2). The answers were given blind to the evaluators by assigning a letter to each LLM, so they were unaware of which LLM they were grading at the time. The correct answer “gold standard,” based on which they were asked to evaluate the answers, was given to the evaluators and was allocated the maximum grade of 10/10. Four weeks after the first evaluation, the answers were graded once again to assess intra-evaluator reliability.

### Statistical analysis

The data were summarized by calculating indices of central tendency (mean and median values) and indices of variability (minimum and maximum values, standard deviations, standard errors of mean values, and coefficient of variation). To test inter-evaluator reliability, that is, if there is a correlation between the grades of the evaluators, *r* and *rho* was calculated. To test reliability, Cronbach’s *alpha* and intraclass correlation coefficient (ICC) were calculated. Furthermore, to test the differences between the grades, Friedman’s test and Wilcoxon’s tests were performed. All statistical analyses were performed with the IBM SPSS (v.29.0) enhanced with the module Exact Tests (for performing the Monte-Carlo simulation method) [21]. The significance level in all hypothesis and testing procedures was predetermined at *a* = 0.05 (*P* ≤ 0.05) [21].

## Results

Table 1 presents the descriptive statistics for the scores given by the two evaluators to the answers provided by the four LLMs, on two different occasions 4 weeks apart, to assess intra-evaluator variability. Both evaluators scored Microsoft Bing Chat’s answers as the best, followed by the answers of ChatGPT 4, Google Bard, and Chat GPT 3.5, on both dates.

**Table 1.** Descriptive statistics for the scores given by the two evaluators to the answers provided by the four LLMs on two different occasions, 4 weeks apart.

	Score 1								Score 2							
	ChatGPT 3.5		ChatGPT 4		Google Bard		Microsoft Bing		ChatGPT 3.5		ChatGPT 4		Google Bard		Microsoft Bing	
Evaluator	1	2	1	2	1	2	1	2	1	2	1	2	1	2	1	2
Min	2	1	3	2	2	2	3	2	2	1	3	1	2	2	3	1
Median	4.0	4.0	5.0	4.5	4.0	4.0	8.0	8.5	4.0	4.0	5.0	4.5	4.0	4.0	8.0	8.5
Max	6	6	7	8	9	9	10	10	5	6	7	8	8	9	10	10
Mean	4.0	3.6	5.1	4.5	4.7	4.7	7.3	7.0	3.8	3.7	4.9	4.4	4.6	4.5	7.2	7.0
SEM	0.37	0.50	0.46	0.61	0.67	0.76	0.73	0.95	0.33	0.50	0.46	0.65	0.60	0.76	0.70	1.04
SD	1.16	1.58	1.45	1.90	2.11	2.41	2.31	3.02	1.03	1.57	1.45	2.07	1.90	2.42	2.20	3.30
CoV	28.9%	43.8%	28.4%	42.2%	44.9%	51.2%	31.7%	43.1%	27.2%	42.4%	29.6%	46.9%	41.2%	53.7%	30.6%	47.1%

CoV, Coefficient of variance; Max, maximum; Min, minimum; SD, standard deviation; SEM, standard error of mean.

The inter-evaluator reliability, that is, the correlation between the scores given by the two evaluators is presented in Table 2. Overall, Pearson’s *r* and Spearman’s *rho* revealed strong and statistically significant correlations between their scores, suggesting that the answers of the four LLMs were corrected in the same way [22, 23]. Similarly, Cronbach’s *alpha* and the ICC suggested high reliability. All Cronbach’s *alpha* values were greater than 0.6 and all ICCs were statistically significant (Table 3). Corroborating evidence was provided by Friedman’s and Wilcoxon’s tests that did not detect, overall, any statistically significant difference between the scores given by the two evaluators on both dates to the answers provided by the four LLMs (Table 4).

**Table 2.** Correlation between the scores given by the two evaluators to the answers provided by the four LLMs on two different occasions (score 1 and 2), 4 weeks apart.

LLM [Evaluator 1–2]	Score 1		Score 2	
	*r* (*P*-value)	*rho* (*P*-value)	*r* (*P*-value)	*rho* (*P*-value)
ChatGPT 3.5	0.671 **(0.034)**	0.673 **(0.033)**	0.645 **(0.044)**	0.608 **(0.040)**
ChatGPT 4	0.700 **(0.024)**	0.900 **(<0.001)**	0.831 **(0.003)**	0.838 **(0.002)**
Google Bard	0.965 **(<0.001)**	0.953 **(<0.001)**	0.970 **(<0.001)**	0.953 **(<0.001)**
Microsoft Bing Chat]	0.924 **(<0.001)**	0.900 **(<0.001)**	0.948 **(<0.001)**	0.953 **(<0.001)**

Statistically significant values in bold.

**Table 3.** Cronbach’s *a* and Intraclass Correlation Coefficient [ICC] for the scores given by the two evaluators to the answers provided by the four LLMs on two different occasions (score 1 and 2), 4 weeks apart, as well as the pooled scores 1 and 2.

LLM	Score 1			Score 2			Pooled Scores 1 & 2		
	Cronbach’s *a*	ICC (*P*-value)		Cronbach’s *a*	ICC (*P*-value)		Cronbach’s *a*	ICC (*P*-value)	
		single	average		single	average		single	average
ChatGPT 3.5	0.78	0.63 **(0.017)**	0.77 **(0.017)**	0.74	0.61 **(0.027)**	0.76 **(0.027)**	0.91	0.74 **(<0.001)**	0.92 **(<0.001)**
ChatGPT 4	0.93	0.83 **(<0.001)**	0.90 **(<0.001)**	0.87	0.76 **(0.002)**	0.86 **(0.002)**	0.96	0.85 **(<0.001)**	0.96 **(<0.001)**
Google Bard	0.97	0.96 **(<0.001)**	0.98 **(<0.001)**	0.97	0.94 **(<0.001)**	0.97 **(<0.001)**	0.99	0.96 **(<0.001)**	0.99 **(<0.001)**
Microsoft Bing	0.94	0.89 **(<0.001)**	0.94 **(<0.001)**	0.93	0.88 **(<0.001)**	0.93 **(<0.001)**	0.97	0.92 **(<0.001)**	0.98 **(<0.001)**

Statistically significant values in bold.

**Table 4.** Wilcoxon’s *p*-value for the scores given by the two evaluators to the answers provided by the four LLMs on each one two different scorings for the intra-evaluator assessment, 4 weeks apart and overall Friedman’s *p*-value for the scores given by the two evaluators for both dates to the answers provided by the four LLMs.

LLM	Wilcoxon’s test		Friedman’s test Pooled Scores 1 and 2
	Score 1	Score 2	
Chat GPT 3.5 [Evaluator 1–2]	0.305	0.792	0.336
Chat GPT 4 [Evaluator 1–2]	0.060	0.212	0.060
Google Bard [Evaluator 1–2]	1.000	0.655	0.667
Microsoft Bing Chat [Evaluator 1–2]	0.417	0.539	0.977

Statistically significant values in bold.

**Figure 1.**
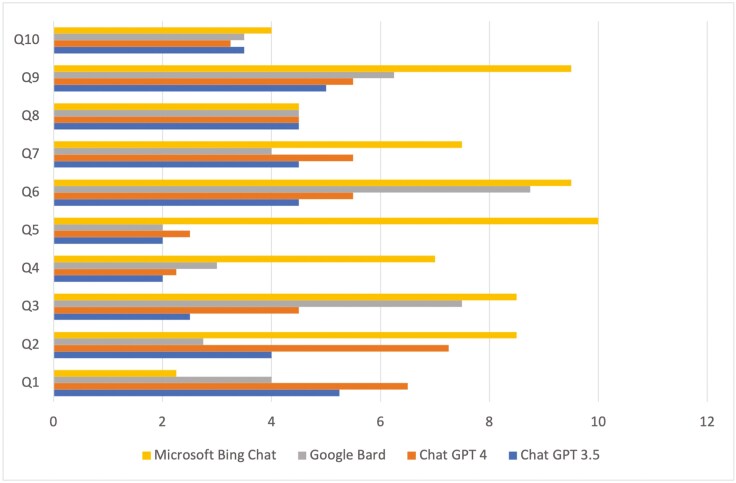
The average scores for the answers to each question provided by the four LLMs.

As a result, an average score was calculated for each LLM from the score of both evaluators given for both dates, to be used in Friedman’s and Wilcoxon tests. Fig. 1 presents the average scores of the answers to each question provided by the four LLMs. Table 5 presents the descriptive statistics for the average scores of the answers provided by the four LLMs. ChatGPT 4 answers were scored as the best, followed by the answers of ChatGPT 3.5, Google Bard, and Microsoft Bing Chat.

**Table 5.** Descriptive statistics for the average scores of the answers provided by the four LLMs.

Average score	Chat GPT 3.5	Chat GPT 4	Google Bard	Microsoft Bing
Min	2.0	2.3	2.0	2.3
Median	4.3	5.0	4.0	8.0
Max	5.3	7.3	8.8	10.0
Mean	3.8	4.7	4.6	7.1
SEM	0.38	0.52	0.69	0.84
SD	1.22	1.66	2.19	2.66
CoV	32.1	35.1	47.3	37.5

CoV, coefficient of variance; Max: maximum; Min: minimum; SD: standard deviation; SEM: standard error of mean.

According to Friedman’s test, statistically significant differences were observed between the average scores of the four LLMs (*P*-value = 0.004). More specifically, a statistically significant difference was noted between the average scores for Chat GPT 3.5 and Chat GPT 4 (*P*-value = 0.011), Chat GPT 3.5 and Microsoft Bing Chat (*P*-value = 0.017), and Google Bard and Microsoft Bing Chat (*P*-value = 0.029) (Table 6). Based on the aforementioned, the LLM answers scoring the best were those of Microsoft Bing Chat (average score = 7.1), followed by ChatGPT 4 (average score = 4.7), Google Bard (average score = 4.6), and finally ChatGPT 3.5 (average score 3.8).

**Table 6.** Wilcoxon’s tests *P*-value for the average scores of the answers provided by the four LLMs.

LLM [average scores]	Wilcoxon’s test
Chat GPT 3.5 vs. Chat GPT 4	**0.011**
Chat GPT 3.5 vs. Google Bard	0.454
Chat GPT 3.5 vs. Microsoft Bing	**0.017**
Chat GPT 4 vs. Google Bard	0.984
Chat GPT 4 vs. Microsoft Bing	0.073
Google Bard vs. Microsoft Bing	**0.029**

Statistically significant values in bold.

## Discussion

While professional and scientific oral healthcare organizations make efforts to incorporate evidence-based approaches into dental clinical practice by formulating and disseminating Clinical Practice Guidelines, persistent adversities, such as rapid advancements in science and technology, outdated guidelines, insufficient evidence, and disruptions to practice workflows, do not facilitate their successful implementation [24]. Despite the risk of “hallucinations” [25], the recent emergence of Generative AI chatbots, capable of generating apparently evidence-based responses to scientific inquiries, could in the future present itself as a potential solution to serve as a dentist’s ‘chairside personal scientific consultant.’ To explore this promising prospect, we assessed the responses of four Language Model chatbots to indicative queries pertaining to various clinically relevant orthodontic topics and clinical decision-making processes encountered in daily practice.

In fact, the LLMs’ descending order in terms of achieved scores was the following: Microsoft Bing Chat came first (average score = 7.1), followed by ChatGPT 4 (average score = 4.7), Google Bard (average score = 4.6), and finally, ChatGPT 3.5 (average score = 3.8). Statistical analysis revealed that Microsoft Bing Chat significantly outperformed ChatGPT-3.5 (*P*-value = 0.017) and Google Bard (*P*-value = 0.029), while ChatGPT 4 demonstrated superior performance compared to ChatGPT 3.5 (*P*-value = 0.011).

The aforementioned assessment rankings may indicate variations in architecture, training data, and performance features among the LLMs considered, impacting their accuracy, relevance, and suitability across different scenarios. Despite the commonality of being language models, these LLMs are built on distinct architectures. For instance, ChatGPT utilizes the GPT (generative pre-trained transformer) architecture, employing a deep learning approach that includes initial training on extensive data followed by fine-tuning for specific tasks. In contrast, Google Bard is founded on Google’s LaMDA (Language Model for Dialogue Application) neural network architecture, prioritizing a better understanding of the context for accurate response generation. In addition, Microsoft Bing AI utilizes various learning models, such as GPT-4, depending on the specific task or application.

Differences in network architectures and variations in the quantity and diversity of training data contribute to Language Models (LLMs) producing unique and varied responses to identical queries, leading to diverse strengths, weaknesses, capabilities, and limitations. Nevertheless, there are notable similarities. In a study conducted by Rudolph *et al.*, which compared the same chatbots as those in the current study for their application in Higher Education, completely different outcomes were noted. ChatGPT-4 secured the top score, followed by ChatGPT-3.5, with Google Bard and Microsoft Bing exhibiting similar rankings [26].

An alternative explanation for discrepancies or inaccuracies in responses, diverging from the established ‘gold standard’, may be attributed to the requisite specificity in prompts for achieving precision. The outputs of Language Models (LLMs) exhibit sensitivity to the level of detail in a question, and certain queries might not have been formulated with sufficient accuracy for the LLMs to comprehend them appropriately [27]. Furthermore, within the domain of medical and dental AI, deficiencies in the representativeness of training datasets, which vary among different LLMs, can lead to inadequacies in generated answers [28]. Dealing with medical and dental inquiries necessitates specialized knowledge and access to high-quality, pertinent scientific data—components potentially lacking in the training data of LLMs, which may not encompass content specific to the respective domains [14]. Additionally, LLMs encounter challenges in grasping intricate relationships between medical conditions and treatment options, hampering their capacity to furnish relevant responses [18].

In the medical field, and specifically in radiology, Rao *et al.* [29] employed a comparable research design to assess ChatGPT’s capability for clinical decision support. This evaluation focused on identifying suitable imaging services for two clinical presentations: breast cancer screening and breast pain. The study compared ChatGPT’s responses with the American College of Radiology (ACR) Appropriateness Criteria, considered as the “gold standard.” In the context of breast cancer screening, ChatGPT scored high in Open-Ended questions, averaging 1.83 out of 2, and demonstrated impressive accuracy in Select All That Apply prompts, with an average of 88.9% correct responses. Notably, for Open-Ended prompts, ChatGPT exhibited more comprehensive reasoning, often providing a detailed rationale for recommending specific imaging modalities in alignment with ACR criteria [29].

In a separate study, Mago and Sharma posed eighty questions on oral and maxillofacial radiology to ChatGPT-3. These questions covered topics such as anatomical landmarks, oral and maxillofacial pathologies, and radiographic features of pathologies. The responses were evaluated by a dentomaxillofacial radiologist. The conclusion drawn was that ChatGPT-3 displayed overall efficiency and could serve as an adjunct when additional information on pathologies is required by an oral radiologist. However, it was emphasized that ChatGPT-3 cannot replace the primary reference source. The limitations highlighted included the model’s inability to provide necessary details and the potential risks of information overload (infodemics) and medical errors associated with its data [27].

In the field of dentistry, Huang *et al.* introduced two principal deployment methods for Language Models (LLMs): automated dental diagnosis and cross-modal dental diagnosis. They thoroughly explored the potential applications of these methods, highlighting the capability of a single LLM, equipped with a cross-modal encoder, to handle multi-source data and engage in sophisticated natural language reasoning for executing complex clinical operations. The researchers further illustrated the potential of a fully automatic Multi-Modal LLM AI system for dentistry clinical applications through presented cases [30]. Giannakopoulos *et al.* assessed how Language Models (LLMs) responded to 20 open-type clinical dentistry-related questions across various disciplines. The findings revealed that among the LLMs examined, ChatGPT-4 emerged as the most proficient in providing answers to the given questions [31]. In the discipline of orthodontics, a recent study concluded that ChatGPT may deliver quality responses to questions pertaining to clear aligners, temporary anchorage devices, and digital imaging in orthodontics [32]. The questions had been previously generated by the LLM and the answers lacked an established objective comparator. The quality of information provided was assessed by five evaluators that exhibited significant divergences in their scores, thus raising issues of potential assessment bias in the employed a crowd score strategy and the evaluation of ChatGPT’s answers.

To the best of the authors’ knowledge, this study marks an initial attempt to evaluate objectively, to the extent possible, the proficiency of multiple Language Models (LLMs) in addressing exclusively orthodontic indicative clinical queries and juxtaposing their responses against a “gold standard” answer. AI models have been proposed to show potential in helping clinicians provide efficient and patient-centered care [13]. In the context of this study, the questions asked were indicative and selected on a basis of clinical relevance and available best evidence in orthodontic literature. Therefore, with a set of 10 questions, it was not possible to cover the entire field of orthodontics. Given the results, it seems that currently LLMs cannot serve as an always reliable source of evidence neither for the patients nor for clinicians who are seeking information online. Specialists’ knowledge cannot be replaced by a LLM’s reply to a query.

Since the aim was to “compare” the LLM answers under, to the extent possible, controlled conditions, follow-up questions were intentionally not asked, in order to avoid introducing bias and other parameters we could not control, such as how many follow-up questions should be asked, what language is used in the follow-up questions, what details should be provided in the follow-up questions etc. Moreover, if the approach of follow-up questions was to be used, comparisons may not have been relevant, since each question could have been dealt differently by each LLM. Subsequent research endeavors are essential to corroborate the findings, which may reflect the current capability of LLMs in responding to orthodontic questions, an aspect that may evolve in the future. Further directions might include a wider range of subjects, asking follow-up questions and evaluating the LLMs’ potential to reproduce reliable answers. Moreover, the applications of LLMs in orthodontic education should be explored as well [33]. This study lays a foundational groundwork for researchers keen on exploring the influence and ramifications of LLMs in dentistry and its branches.

## Conclusions

Language models (LLMs) unquestionably show promise in supporting evidence-based orthodontics. Nonetheless, their current limitations introduce a potential hazard of making inaccurate healthcare decisions if utilized without due diligence. Therefore, it is vital not to allow these tools to replace the orthodontist’s essential critical thinking and extensive subject knowledge. To guarantee their successful incorporation into practice, it is imperative to undertake additional research, conduct clinical validation, and make enhancements to the models. Clinicians need to recognize the limitations of LLMs, as their unwise application may adversely affect patient care.

## Supplementary Material

cjae017_suppl_Supplementary_Table_1

cjae017_suppl_Supplementary_Table_2

## Data Availability

The data underlying this article are available in the article and in its online supplementary material.
