# Supplementary material for: Evidence-based potential of generative artificial intelligence large language models in orthodontics: a comparative study of ChatGPT, Google Bard, and Microsoft Bing
Source: Eur J Orthod. 2024 Apr 13;48(1):cjae017. doi: 10.1093/ejo/cjae017 (PMC12810200; doi:10.1093/ejo/cjae017)
Supplement: cjae017_suppl_Supplementary_Table_1 [file cjae017_suppl_supplementary_table_1.docx]

**Supplementary Table 1.**

| **Questions/discipline** | **ChatGPT 3.5**  [Introducing ChatGPT (openai.com)](https://openai.com/blog/chatgpt) | **ChatGPT 4**  <https://chat.openai.com/?model=gpt-4> | **Bard**  [‎Δοκιμάστε το Bard, ένα πείραμα AI από την Google](https://bard.google.com/) | **Bing chat version**  [Bing AI - Αναζήτηση](https://www.bing.com/search?form=NTPCHB&q=Bing+AI&showconv=1) | **Evidence (literature)** |
| --- | --- | --- | --- | --- | --- |
| 1. Does orthodontic treatment affect the airway function? | Orthodontic treatment can potentially affect airway function, but the extent and direction of this impact can vary depending on several factors, including the type of orthodontic treatment, the patient's individual anatomy, and the goals of treatment. Here are some key points to consider:  1. **Expansion of the Dental Arch**: Orthodontic treatments like palatal expansion can create more space in the oral cavity. This can potentially improve airflow and alleviate certain breathing problems, particularly in cases where narrow dental arches contribute to airway constriction.  2. **Orthognathic Surgery**: In some cases, orthodontic treatment is combined with orthognathic (jaw) surgery to correct severe jaw discrepancies. This surgery can have a significant impact on the airway, especially if it involves repositioning the upper or lower jaw to improve the alignment of the teeth and facial structures.  3. **Obstructive Sleep Apnea (OSA)**: Orthodontic treatment may be considered as part of a multidisciplinary approach to manage obstructive sleep apnea. For example, devices like mandibular advancement devices (MADs) can be used to reposition the lower jaw and tongue to help keep the airway open during sleep.  4. **Retention and Relapse**: After orthodontic treatment, the use of retainers is essential to maintain the newly aligned teeth. However, certain types of retainers, such as removable ones that cover the palate, may potentially affect airflow temporarily.  5. **Individual Anatomy**: Airway function is highly individual, and the impact of orthodontic treatment can vary widely among patients. Some individuals may experience improved airflow as a result of orthodontic treatment, while others may not notice significant changes.  It's important to note that while orthodontic treatment can have potential effects on airway function, the primary goal of orthodontics is typically to correct dental and skeletal malocclusions (misalignments) and improve the aesthetics and function of the teeth and jaws. If concerns about airway function are a primary issue, it's advisable to consult with a multidisciplinary team that includes an orthodontist, oral and maxillofacial surgeon, and a sleep medicine specialist to determine the most appropriate treatment approach.  Additionally, advancements in orthodontic techniques and technologies are continually evolving, and some orthodontists may incorporate airway considerations into treatment planning when appropriate. It's essential to have a thorough discussion with your orthodontist about your specific concerns and goals for treatment to ensure that the potential impact on airway function is considered as part of your overall orthodontic plan. | Orthodontic treatment can potentially influence the airway function, although the precise nature and extent of this influence can vary depending on the type of orthodontic intervention, the specific anatomical considerations of the patient, and other factors. Here are some ways in which orthodontic treatment might affect airway function:   1. **Extraction vs. Non-extraction Orthodontics**: There has been some debate about whether removing permanent teeth (especially premolars) to alleviate crowding can affect the size or function of the airway. Some believe that extraction can lead to a posterior positioning of the tongue and possibly compromise the airway. However, research in this area has yielded mixed results, and there isn’t a universal consensus. 2. **Mandibular Advancement**: Orthodontic treatments that involve moving the lower jaw forward (like functional appliances or orthognathic surgery for individuals with retrognathic mandibles) can increase the size of the oropharyngeal airway. This might help in alleviating symptoms of obstructive sleep apnea in some individuals. 3. **Expansion of the Palate**: Rapid palatal expanders, which widen the upper jaw, can increase the width of the nasal floor and potentially improve nasal breathing. Some studies have shown an increase in nasal airway dimensions and improved breathing after palatal expansion. 4. **Orthodontic Treatment and Sleep Apnea**: There's a growing interest in the potential for orthodontic and orthopedic treatments to address issues related to sleep apnea, especially in children. For example, children with enlarged adenoids or tonsils can sometimes present with constricted dental arches and mouth breathing. In some cases, a combination of tonsil and/or adenoid removal and orthodontic expansion can improve airway symptoms. 5. **Treatment of Vertical Growth Patterns**: Patients with a downward and backward growth pattern of the jaws might have a compromised airway. Certain orthodontic and orthopedic treatments aim to redirect this growth to create a more favorable jaw relationship and potentially benefit the airway. 6. **Changes in Tongue Position**: Some orthodontic treatments can influence the resting position of the tongue. Since the tongue plays a vital role in airway patency, any changes to its position can potentially affect the airway.   It's worth noting that while orthodontic treatment can influence the airway, it is not always the primary solution for airway problems. Additionally, the individual variability is considerable; what works for one patient might not work for another. Proper diagnosis, often involving a multidisciplinary approach with specialists like ENTs, sleep physicians, and orthodontists, is crucial for determining the best treatment plan for any airway-related concerns. | The answer to this question is not straightforward, as the effects of orthodontic treatment on airway function can vary depending on the individual patient and the specific treatment plan.  In general, orthodontic treatment that involves the retraction of the front teeth (such as with braces) can narrow the upper airway. This is because the tongue needs more space to rest behind the front teeth, and if the front teeth are retracted, the tongue may not have enough room and may collapse into the airway.  However, other types of orthodontic treatment, such as widening the upper jaw (with a palatal expander) or moving the molars back (with headgear), can actually improve airway function by increasing the space in the airway.  Ultimately, the best way to determine whether orthodontic treatment will affect your airway function is to talk to your orthodontist. They will be able to assess your individual situation and recommend the best treatment plan for you.  Here are some of the factors that your orthodontist will consider when evaluating the potential impact of orthodontic treatment on your airway function:   - Your skeletal Class: This refers to the relationship between your upper and lower jaws. People with a Class II skeletal relationship (the upper jaw is more forward than the lower jaw) are more likely to have airway problems than people with a Class I skeletal relationship (the upper and lower jaws are in the correct relationship). - Your dental malocclusion: This refers to the alignment of your teeth. People with a Class III malocclusion (the lower jaw is more forward than the upper jaw) are also more likely to have airway problems than people with a Class I malocclusion. - Your body mass index (BMI): People with a higher BMI are more likely to have airway problems, regardless of their skeletal Class or dental malocclusion. - Your symptoms: If you have any symptoms of airway obstruction, such as snoring, sleep apnea, or difficulty breathing, your orthodontist will need to take these into account when planning your treatment.   If you are concerned about the potential impact of orthodontic treatment on your airway function, be sure to talk to your orthodontist. They can help you understand the risks and benefits of different treatment options and recommend the best plan for you. | Hello, this is Bing. Orthodontic treatment can affect the airway function in different ways, depending on the type and goal of the treatment. Some forms of orthodontic treatment, such as airway orthodontics, aim to improve the function of the airway by expanding the oral cavity and correcting the jaw position123. Other forms of orthodontic treatment, such as premolar extractions, may have an indirect effect on the airway by changing the skeletal and dental features4. However, the size of the airway does not necessarily reflect its function5. The effects of orthodontic treatment on the airway may vary depending on the individual’s anatomy and physiology6. Are you looking for more information on a specific type of orthodontic treatment?  Learn more:  1. helloortho.com  2. functionfirstinfo.com  3. truealignment.co.nz  4. meridian.allenpress.com  5. speareducation.com  6. intechopen.com  +3 more | Behrents RG, Shelgikar AV, Conley RS, Flores-Mir C, Hans M, Levine M, McNamara JA, Palomo JM, Pliska B, Stockstill JW, Wise J, Murphy S, Nagel NJ, Hittner J. Obstructive sleep apnea and orthodontics: An American Association of Orthodontists White Paper. Am J Orthod Dentofacial Orthop. 2019 Jul;156(1):13-28.e1. doi: 10.1016/j.ajodo.2019.04.009. PMID: 31256826.  Summary of evidence:  Conventional orthodontic treatment has never been proven to be an etiologic factor in the development of OSA.  Overall, it can be stated that existing evidence in the literature does not support the notion that arch constriction or retraction of the anterior teeth facilitated by dental extractions, and which may (or may not) be the objective of orthodontic treatment, has a detrimental effect on respiratory function.  No orthodontic treatments have been shown to cause or increase the likelihood of OSA. Rather, some forms of orthodontic treatment have been shown to be important in the treatment of OSA.  GRADE: 10/10 |
| 2. Is early orthodontic treatment in two phases for children with prominent upper teeth, more beneficial compared to treatment that is provided in one phase in adolescence? | Whether early orthodontic treatment in two phases for children with prominent upper teeth is more beneficial compared to treatment provided in one phase during adolescence depends on the individual patient's needs and the specific orthodontic issues they are facing. There is no one-size-fits-all answer, as the optimal treatment approach varies from case to case. Here are some considerations for both approaches:  **Early Orthodontic Treatment in Two Phases (Phase I and Phase II):**  1. **Phase I (Interceptive Phase):** This phase typically begins when a child still has a mix of baby and permanent teeth, usually around the ages of 7-10. It aims to address specific orthodontic issues early, such as severe crowding, significant overbites or underbites, or prominent upper teeth.  2. **Benefits:**  - Addresses critical issues early, potentially reducing the severity of problems that might worsen over time.  - May improve facial aesthetics and self-esteem in cases of prominent upper teeth.  - Can create more favorable conditions for the eruption of permanent teeth.  - May reduce the need for more extensive treatment in adolescence.  3. **Drawbacks:**  - Requires two phases of treatment, which can be more time-consuming and costly.  - The patient may need to wear braces or other appliances at a young age.  **Orthodontic Treatment in One Phase in Adolescence:**  1. **Single-Phase Treatment:** This approach usually begins in adolescence, typically between the ages of 11-15 when most permanent teeth have erupted. It aims to address orthodontic issues comprehensively in one phase.  2. **Benefits:**  - Simplifies treatment into a single phase, which can be more convenient.  - May avoid the need for two separate phases of treatment.  - Adolescents are often more cooperative with orthodontic treatment than younger children.  3. **Drawbacks:**  - Some orthodontic issues, if severe, may become more challenging to correct if not addressed earlier.  - Prominent upper teeth could lead to aesthetic and functional concerns during the child's formative years.  The decision between these two approaches should be made after a thorough evaluation by an orthodontist. Factors to consider include the nature and severity of the orthodontic problems, the patient's age and growth pattern, and the potential impact on oral health, aesthetics, and overall well-being.  In some cases, early orthodontic treatment may be recommended to address critical issues, such as prominent upper teeth, while other problems may be addressed in a single phase during adolescence. It's essential to have a comprehensive discussion with your orthodontist to determine the most appropriate treatment plan for your child's specific needs and circumstances. | Early orthodontic intervention, also referred to as two-phase treatment, is often recommended for children with prominent upper teeth (often called Class II malocclusion or overjet). The goal of early intervention is to address skeletal discrepancies, dental malocclusions, and psychosocial concerns before all permanent teeth erupt.  Here's a breakdown of the potential benefits and considerations associated with early two-phase treatment versus one-phase treatment during adolescence:  Two-Phase Treatment:   1. Benefits:    - Early Correction: Prominent upper teeth can be at risk of trauma, especially in active children. Early intervention may reduce this risk.    - Growth Modification: Early treatment can take advantage of the patient's growth to correct skeletal discrepancies.    - Psychosocial Reasons: Prominent front teeth can sometimes be a source of teasing or reduced self-esteem in children. Early correction can address these concerns.    - Potentially Easier Treatment: Some problems might be easier to address while the jaw is still growing and might result in less invasive procedures later. 2. Considerations:    - Duration: Two-phase treatment often means a longer total time in orthodontic appliances, as there's an initial phase of treatment during childhood followed by a period of rest and then a second phase during adolescence.    - Cost: Two-phase treatment can be more expensive than one-phase treatment.    - Not Always Necessary: Some orthodontic concerns can wait and be effectively addressed in adolescence. Not all children with prominent upper teeth need early intervention.   One-Phase Treatment in Adolescence:   1. Benefits:    - Shorter Treatment Time: Only one phase of orthodontic intervention is needed, which means a potentially shorter time in braces or other orthodontic appliances.    - Cost-effective: It might be less expensive overall than two-phase treatment. 2. Considerations:    - Missed Growth Opportunity: By waiting until adolescence, some opportunities to guide jaw growth may be missed, possibly necessitating more invasive procedures or extractions.    - Potential for Trauma: Prominent upper teeth remain at risk of trauma until corrected.   Conclusion:  The decision between early two-phase treatment and one-phase treatment in adolescence should be based on an individual's specific needs. Factors to consider include the degree of skeletal discrepancy, risk of trauma, psychosocial concerns, patient and parent preferences, and the recommendation of the orthodontist. Always consult with a qualified orthodontist to get a personalized recommendation based on the specific circumstances and needs of the child. | Yes, early orthodontic treatment in two phases for children with prominent upper teeth can be more beneficial than treatment that is provided in one phase in adolescence.  Here are some of the benefits of early orthodontic treatment:   - It can help to prevent the development of more serious problems later on. For example, prominent upper teeth can put increased pressure on the jaw joint, which can lead to pain and problems with chewing. Early treatment can help to relieve this pressure and prevent these problems from developing. - It can help to improve the alignment of the teeth and jaws, which can make it easier to clean the teeth and reduce the risk of tooth decay and gum disease. - It can improve the child's self-esteem and confidence. Children with prominent upper teeth may be self-conscious about their smile, and early treatment can help to improve their appearance and make them feel better about themselves.   The two phases of early orthodontic treatment are typically:   - Phase 1: This is usually done when the child is between the ages of 7 and 11 years old. The goal of this phase is to correct the underlying skeletal problems that are causing the prominent upper teeth. This may involve using appliances such as headgear or palatal expanders. - Phase 2: This is usually done when the child is in early adolescence, between the ages of 12 and 16 years old. The goal of this phase is to fine-tune the alignment of the teeth and jaws. This may involve using braces or retainers.   It is important to note that not all children with prominent upper teeth need early orthodontic treatment. The decision of whether or not to treat a child with early orthodontic treatment is made on a case-by-case basis by the orthodontist.  If you are concerned about your child's prominent upper teeth, it is important to talk to your orthodontist. They can assess your child's individual situation and recommend the best treatment plan for them. | According to the evidence, **early orthodontic treatment in two phases** for children with prominent upper teeth is more beneficial than treatment in one phase in adolescence, because it **significantly reduces the incidence of damage to the upper incisor teeth**¹²³. However, other factors such as cost, duration, compliance and aesthetics may also influence the decision to choose one type of treatment over another. Do you have any specific concerns or preferences regarding orthodontic treatment?  Source: Conversation with Bing, 8/24/2023  (1) Orthodontic treatment for prominent upper front teeth (Class II .... https://www.cochranelibrary.com/cdsr/doi/10.1002/14651858.CD003452.pub3/abstract.  (2) Highlighted review: Orthodontic treatment for prominent upper front .... https://oralhealth.cochrane.org/news/highlighted-review-orthodontic-treatment-prominent-upper-front-teeth-class-ii-malocclusion.  (3) Orthodontic treatment for prominent upper front teeth in children. https://www.cochrane.org/CD003452/ORAL_orthodontic-treatment-prominent-upper-front-teeth-children. | Batista KBSL, Thiruvenkatachari B, Harrison JE, O'Brien KD.  Orthodontic treatment for prominent upper front teeth (Class II malocclusion) in children and adolescents. Cochrane Database of Systematic Reviews 2018, Issue 3. Art. No.: CD003452.  Summary of evidence:  The evidence suggests that providing orthodontic early treatment to children with prominent upper front teeth reduces the incidence of damage to upper incisor teeth significantly (middle four teeth at the top) as compared to treatment that is provided in one phase in adolescence. There are no other advantages of providing a two-phase treatment (i.e. between age seven to 11 years and again in adolescence) compared to treatment in one phase in adolescence.  GRADE: 10/10 |
| 3. Do light vibrational forces reduce the duration of orthodontic treatment? | Light vibrational forces, when applied appropriately, may help accelerate orthodontic tooth movement and potentially reduce the duration of orthodontic treatment. This concept is often referred to as "accelerated orthodontics" or "vibrational orthodontics." Here's how it works:  1. **Mechanical Vibrations:** Vibrational forces involve the application of gentle, controlled vibrations to the teeth and surrounding bone. These vibrations can be applied using specialized devices designed for orthodontic treatment.  2. **Biological Response:** The application of these vibrations stimulates the bone remodeling process around the tooth roots. This stimulation can lead to faster tooth movement because the bone can adapt more quickly to the new tooth positions.  3. **Reduced Discomfort:** Patients often report less discomfort and pain during orthodontic treatment when vibrational forces are used. This can improve overall patient comfort throughout the treatment process.  4. **Potential Reduction in Treatment Time:** Accelerated orthodontic techniques, including vibrational forces, have the potential to shorten the overall duration of orthodontic treatment. However, the extent of the reduction in treatment time can vary among individuals.  It's important to note that while vibrational orthodontics may offer some benefits, it may not be suitable for every patient or every type of orthodontic issue. The suitability of this technique depends on factors such as the complexity of the orthodontic case and the patient's overall oral health.  Before considering vibrational orthodontics or any other accelerated orthodontic technique, it's essential to consult with an experienced orthodontist. They will assess your specific orthodontic needs and recommend the most appropriate treatment plan. Additionally, orthodontic treatment involves many factors beyond just tooth movement, such as maintaining proper bite alignment and achieving long-term stability, which should also be considered when determining the overall treatment duration. | As of my last training data in September 2021, the use of vibrational devices in orthodontics is a topic that has garnered interest as a potential means to accelerate tooth movement and thus reduce the duration of orthodontic treatment.  The rationale behind this approach is that the application of vibrational forces might stimulate cellular activity in the periodontal ligament and surrounding bone, which could potentially facilitate faster tooth movement when orthodontic forces (like those from braces or aligners) are applied.  However, the available research on this topic has been mixed:   1. **Positive Results**: Some studies have suggested that vibrational devices might provide a benefit in accelerating tooth movement. Patients using these devices in conjunction with regular orthodontic appliances might experience slightly quicker treatment times or reduced discomfort. 2. **Neutral or Negative Results**: Other studies haven't found a significant difference in the rate of tooth movement between patients using vibrational devices and those not using them. Additionally, some reviews have pointed out that while there might be an initial acceleration in tooth movement, the overall duration of orthodontic treatment might not be significantly impacted. 3. **Comfort and Compliance**: Another angle of research has looked at whether vibrational devices can help reduce the pain or discomfort associated with orthodontic tooth movement. Some patients feel that these devices reduce their discomfort, which could indirectly improve compliance with orthodontic treatment and potentially affect treatment duration.   It's important to note that the quality of research varies, and more rigorous randomized controlled trials are needed to draw definitive conclusions about the effectiveness of vibrational devices in orthodontics.  If you or someone you know is considering using vibrational devices as part of orthodontic treatment, it would be best to discuss it with an orthodontist. They can provide insights into the latest research, potential benefits, and the appropriateness of such devices for the individual's specific case. | The current evidence suggests that light vibrational forces do not reduce the duration of orthodontic treatment.  There have been a number of studies that have investigated the use of light vibrational forces to accelerate orthodontic tooth movement. However, the results of these studies have been mixed, and no clear benefit of light vibrational forces has been established.  One study found that light vibrational forces could reduce the duration of orthodontic treatment by about 2 weeks. However, other studies have found no significant difference in the duration of treatment between groups that received light vibrational forces and groups that did not.  It is important to note that the studies that have been conducted on light vibrational forces have been small and short-term. More research is needed to determine whether light vibrational forces can be used to safely and effectively reduce the duration of orthodontic treatment.  If you are considering using light vibrational forces to accelerate your orthodontic treatment, it is important to talk to your orthodontist. They can assess your individual situation and recommend the best treatment plan for you.  Here are some other factors that can affect the duration of orthodontic treatment:   - The severity of the malocclusion: The more severe the malocclusion, the longer the treatment will take. - The patient's age: Children tend to respond to orthodontic treatment more quickly than adults. - The patient's compliance: Patients who are more compliant with their treatment (wearing their braces or retainers as directed) will typically have shorter treatment times. - The type of orthodontic appliance used: Some appliances, such as Invisalign, can be removed by the patient, which can make them more comfortable to wear and may lead to shorter treatment times.   If you are concerned about the duration of your orthodontic treatment, be sure to talk to your orthodontist. They can help you understand the factors that may affect your treatment time and recommend the best treatment plan for you. | The evidence suggests that **light vibrational forces** do not have a significant effect on **reducing the duration of orthodontic treatment** compared to a control¹³⁴. The mean difference was 0.6 months, which is not clinically relevant¹. However, some studies have claimed that light vibrational forces may have other benefits, such as accelerating tooth movement or alleviating pain, but these results are not consistent or conclusive². Are you interested in learning more about these potential benefits?  Source: Conversation with Bing, 8/24/2023  (1) A massive Cochrane review on the non-surgical methods to speed up tooth .... https://kevinobrienorthoblog.com/a-massive-cochrane-review-on-non-surgical-methods-to-speed-up-tooth-movement/.  (2) Additional, non-surgical treatments for accelerating tooth ... - Cochrane. https://www.cochrane.org/CD010887/ORAL_additional-non-surgical-treatments-accelerating-tooth-movement-dental-patients-being-treated-fixed.  (3) Library Cochrane. https://www.cochranelibrary.com/cdsr/doi/10.1002/14651858.CD010887.pub2/pdf/CDSR/CD010887/CD010887_abstract.pdf.  (4) effectiveness of supplemental vibrational force on enhancing .... https://academic.oup.com/ejo/article/41/5/502/5486632. | El-Angbawi A, McIntyre G, Fleming PS, Bearn D. Non-surgical adjunctive interventions for accelerating tooth movement in patients undergoing orthodontic treatment. Cochrane Database Syst Rev. 2023 Jun 20;6(6):CD010887. doi: 10.1002/14651858.CD010887.pub3. PMID: 37339352; PMCID: PMC10281004.  Summary of evidence:  There is no additional benefit of light vibrational forces or photobiomodulation for reducing the duration of orthodontic treatment..  GRADE: 10/10 |
| 4. How long should one wait to go for surgical exposure after an interceptive intervention for palatally displaced permanent canine? | The timing for surgical exposure of a palatally displaced permanent canine after an interceptive intervention can vary depending on the individual patient's dental development and the specific circumstances of the case. Here are some general guidelines:  1. **Assessment and Monitoring:** After an interceptive intervention, such as a palatal expansion or space maintenance appliance, the patient's dental development should be closely monitored by an orthodontist. X-rays and clinical examinations are typically used to assess the position and eruption progress of the palatally displaced permanent canine.  2. **Timing of Surgical Exposure:** The timing for surgical exposure and orthodontic bracket placement on the impacted canine is typically determined based on the following factors:    - **Eruption Progress:** If the canine is making good progress toward erupting into its proper position after the interceptive treatment, the orthodontist may choose to delay surgical exposure to allow for natural eruption.    - **Patient's Age and Growth:** The patient's age and growth pattern are important considerations. In some cases, it may be advisable to wait until the patient has reached a certain age or stage of dental development before proceeding with surgery.  - **Severity of Impaction:** The severity of the impaction and the position of the impacted canine in relation to nearby teeth will also influence the timing of surgical exposure. Severe impactions or canines positioned deep within the palate may require earlier surgical intervention.  3. **Orthodontic Planning:** The orthodontist will work in coordination with the oral surgeon to plan the surgical exposure procedure and subsequent orthodontic treatment. This includes determining the best time to expose the impacted tooth, attach an orthodontic bracket to it, and begin guiding it into its correct position in the dental arch.  4. **Individualized Treatment:** It's important to emphasize that the timing of surgical exposure should be individualized for each patient based on their unique dental and skeletal characteristics. There is no one-size-fits-all answer, and decisions should be made in consultation with an experienced orthodontist and oral surgeon.  In summary, the timing of surgical exposure for a palatally displaced permanent canine after an interceptive intervention is a carefully considered decision that depends on multiple factors. Regular follow-up appointments with an orthodontist are essential to monitor the progress of the impacted tooth and determine the optimal timing for surgical intervention if necessary. The goal is to achieve proper alignment and eruption of the impacted canine while minimizing treatment duration and complications. | I'm not a dentist, but in general, the management of palatally displaced canines (PDCs) involves a combination of interceptive and surgical interventions, depending on the specific clinical scenario. The goal is to facilitate the proper eruption of the impacted canine or to place it in a position where it can be more predictably brought into the arch orthodontically.  When an interceptive intervention (like extraction of the primary canine or other space management) has been done to aid in the eruption of a palatally displaced permanent canine, the waiting period before considering surgical exposure can vary based on several factors:   1. **Age of the Patient:** The younger the patient, the more potential there is for the tooth to redirect and erupt on its own after interceptive measures. Generally, for young patients (below 13 years), one might wait longer before intervening surgically. 2. **Position of the Canine:** The position and angulation of the canine on radiographs can give information about its potential to erupt. If the tooth is more horizontally positioned or if it's positioned quite far palatally, there might be a lesser chance of it erupting without surgical assistance. 3. **Radiographic Assessment:** Sequential radiographs can provide information about any movement of the canine following interceptive measures. If the canine is seen to be moving in a favorable direction, a decision might be made to wait and monitor. On the other hand, if there's no discernible movement over a certain period, surgical exposure might be considered. 4. **Space Assessment:** Ensuring there's adequate space in the dental arch for the canine to erupt is crucial. If space is insufficient even after interceptive measures, surgical exposure and orthodontic alignment might be indicated sooner. 5. **Expert Opinion:** Different orthodontists or oral surgeons might have different thresholds for when they believe surgical exposure should be done. It's always a balance between giving the tooth time to erupt on its own versus waiting too long and potentially complicating treatment.   As a general timeframe, after interceptive measures are taken, many clinicians will monitor the situation with periodic radiographs for 6 months to a year (or sometimes even longer for younger patients) to observe if the canine shows signs of spontaneous eruption. If there's no evidence of progress after this period, a surgical exposure combined with orthodontic traction may be considered.  Always consult with an orthodontist and/or an oral surgeon to make the best clinical decision for the specific patient in question. | The decision of when to go for surgical exposure after an interceptive intervention for palatally displaced permanent canine depends on a number of factors, including the position of the tooth, the age of the patient, and the patient's overall health.  In general, it is recommended to wait at least 6 months after the interceptive intervention before considering surgical exposure. This gives the tooth time to start to erupt on its own. If the tooth has not erupted after 6 months, or if it is not erupting in the correct direction, then surgical exposure may be necessary.  The age of the patient is also a factor to consider. Younger patients are more likely to have successful outcomes with surgical exposure than older patients. This is because the bone is more pliable in younger patients, which makes it easier to move the tooth into the correct position.  The patient's overall health is also a factor to consider. Patients with certain medical conditions, such as diabetes or heart disease, may be at increased risk of complications from surgery. In these cases, the orthodontist may recommend waiting until the patient's health improves before proceeding with surgery.  Ultimately, the decision of when to go for surgical exposure is made on a case-by-case basis by the orthodontist. They will take into account all of the factors involved and recommend the best course of treatment for the individual patient.  Here are some additional things to consider when deciding whether or not to proceed with surgical exposure:   - The likelihood of the tooth erupting on its own without surgery. - The risk of complications from surgery. - The patient's age and overall health. - The patient's wishes and preferences.   If you are considering surgical exposure for a palatally displaced permanent canine, it is important to talk to your orthodontist. They can help you understand the risks and benefits of surgery and recommend the best course of treatment for you. | The timing of surgical exposure after interceptive intervention for palatally displaced canines (PDCs) may depend on several factors, such as the degree of displacement, the eruption status, the surgical technique and the orthodontic treatment plan. However, some studies have suggested that **surgical exposure should be performed as soon as possible after interceptive intervention** to avoid further complications and reduce treatment time¹². The average treatment time for surgical exposure of PDCs ranges from **18 to 24 months**¹². However, in some cases, surgical exposure may not be required if the canine erupts spontaneously after interceptive intervention³. Do you have a specific case or scenario in mind?  Source: Conversation with Bing, 8/24/2023  (1) Open vs closed surgical exposure of palatally displaced canines: a .... https://academic.oup.com/ejo/article/43/5/487/6296675.  (2) Closed vs open surgical exposure of palatally displaced canines .... https://academic.oup.com/ejo/article/40/6/626/5129098.  (3) Management of the palatally displaced canine \| Clinical ... - Springer. https://link.springer.com/article/10.1007/s41894-018-0039-z. | Benson PE, Atwal A, Bazargani F, Parkin N, Thind B. Interventions for promoting the eruption of palatally displaced permanent canine teeth, without the need for surgical exposure, in children aged 9 to 14 years. Cochrane Database Syst Rev. 2021 Dec 30;12(12):CD012851. doi: 10.1002/14651858.CD012851.pub2. PMID: 34967448; PMCID: PMC8717471.  Summary of evidence:  If the unerupted PDC has not erupted by 18 months following any intervention then the probability of eruption may be low and alternative methods of management should be considered.  GRADE: 10/10 |
| 5. What is the best orthodontic treatment for posterior crossbites? | The most appropriate orthodontic treatment for posterior crossbites depends on the specific nature and severity of the crossbite, the patient's age, and various other factors. There are several treatment options available, and the best one will be determined by the orthodontist after a thorough evaluation. Here are common treatment approaches for posterior crossbites:  1. **Orthodontic Treatment:**  - **Braces:** Traditional braces can be used to correct posterior crossbites. Brackets and wires are adjusted to gradually move the teeth into their proper positions.  - **Invisalign or Clear Aligners:** Clear aligner systems like Invisalign may also be suitable for treating posterior crossbites, especially if they are relatively mild.  2. **Palatal Expansion:** This treatment is often used in growing children and adolescents when the upper jaw (maxilla) is too narrow, causing a posterior crossbite. A palatal expander is a device that widens the upper jaw to create more space for the teeth. It can be fixed or removable, depending on the patient's age and needs.  3. **Rapid Maxillary Expansion (RME):** RME is a type of palatal expansion that is done relatively quickly (typically over a period of weeks) to correct a narrow upper jaw. It's often used in children with developing dental arches.  4. **Surgical Options:** In some cases of severe posterior crossbites, especially in adults, surgical procedures may be necessary to correct the jaw misalignment. This can involve orthognathic surgery to reposition the upper or lower jaw to achieve proper alignment.  5. **Functional Appliances:** Functional appliances are sometimes used in cases where there is a skeletal component to the crossbite. These appliances help stimulate growth and guide the jaws into proper alignment.  6. **Orthodontic Retainers:** After the crossbite is corrected, orthodontic retainers are often used to maintain the corrected position of the teeth and prevent relapse.  The choice of treatment will depend on factors such as the age of the patient, the severity and cause of the crossbite, and the overall goals of orthodontic treatment (e.g., aesthetics, functional improvement). It's crucial to consult with an experienced orthodontist who can evaluate your specific case and recommend the most appropriate treatment plan.  Early intervention is often preferred for posterior crossbites, especially in children, as it can help prevent more significant problems from developing and make treatment more straightforward. However, successful treatment outcomes can be achieved in patients of all ages with the right approach and expertise. | I am not a dentist, but in general, the best orthodontic treatment for a posterior crossbite often depends on the specific nature of the crossbite, its cause, the age of the patient, and other individual factors. Posterior crossbites can result from a combination of skeletal (jaw-based) and dental (tooth-based) discrepancies.  Some potential treatments for posterior crossbites include:   1. **Quad Helix Appliance**: This is a fixed appliance that is cemented onto the back molars. It is primarily used in children and is designed to widen the upper jaw, allowing the upper teeth to fit outside the lower teeth. 2. **Rapid Palatal Expander (RPE)**: This is another fixed appliance that is used to widen the upper jaw in growing children. It is typically worn for several months and is adjusted by turning a screw in the center of the appliance, which applies pressure and gradually expands the palate. 3. **Removable Expander**: Some cases may be treated with a removable expansion appliance, although it might not be as effective as fixed appliances for certain situations. 4. **Braces**: Traditional braces can be used to correct a dental crossbite once the skeletal discrepancy has been managed. In some cases, braces alone might suffice, especially if the crossbite is minor or is primarily tooth-based. 5. **Clear Aligners**: Brands like Invisalign or other clear aligner systems can sometimes be used to treat minor crossbites, especially in adults. 6. **Tooth Reshaping**: In cases where a tooth interferes with the bite due to its shape or size, minor reshaping of the tooth might be a solution. 7. **Jaw Surgery**: In severe cases, especially where there's a significant skeletal discrepancy, orthognathic surgery might be recommended. This is more commonly an option for adults whose jaw growth has completed. 8. **Early Intervention**: In young children, the use of certain appliances or strategies like guiding eruption can prevent a crossbite from developing or worsening.   It's essential to consult with an orthodontist or dentist to get a comprehensive diagnosis and recommendation tailored to individual needs. They can provide detailed information on the best treatment options and expected outcomes for specific cases. | The best orthodontic treatment for posterior crossbites depends on a number of factors, including the severity of the crossbite, the patient's age, and the patient's overall health.  In general, there are two main types of orthodontic treatment for posterior crossbites:   - Palatal expansion: This is a treatment that widens the upper jaw. This can be done with a fixed appliance called a quad-helix or with a removable appliance called an expansion plate. - Functional appliances: These are appliances that help to correct the bite by changing the way the teeth and jaws function. Examples of functional appliances include the Herbst appliance and the Twin-Block appliance.   The decision of which type of treatment is best for a particular patient is made by the orthodontist. They will take into account all of the factors involved and recommend the best course of treatment for the individual patient.  Here are some of the factors that the orthodontist will consider when making a treatment recommendation:   - The severity of the crossbite: The more severe the crossbite, the more likely it is that surgery will be needed. - The patient's age: Children tend to respond to orthodontic treatment more quickly than adults. - The patient's overall health: Patients with certain medical conditions, such as diabetes or heart disease, may be at increased risk of complications from orthodontic treatment. - The patient's wishes and preferences: Some patients may prefer to have a less visible appliance, while others may be more concerned about the length of treatment.   If you are considering orthodontic treatment for a posterior crossbite, it is important to talk to your orthodontist. They can help you understand the different treatment options and recommend the best course of treatment for you.  Here are some additional things to consider when choosing an orthodontic treatment for posterior crossbite:   - The cost of treatment: Orthodontic treatment can be expensive, so it is important to factor in the cost when making a decision. - The time commitment: Orthodontic treatment can take several years, so it is important to be prepared for the time commitment. - The potential side effects: Orthodontic treatment can have some side effects, such as tooth sensitivity and gum irritation. It is important to be aware of the potential side effects before starting treatment.   If you are concerned about the cost, time commitment, or potential side effects of orthodontic treatment, be sure to talk to your orthodontist. They can help you understand the risks and benefits of different treatment options and recommend the best course of treatment for you. | According to a Cochrane review¹², **orthodontic treatment with quad‐helix (fixed) or expansion plates (removable)** is effective for correcting posterior crossbite in children. Quad‐helix is probably even more effective than expansion plates. For adolescents, there is probably no difference between Hyrax and Haas for posterior crossbite correction¹²³. However, the treatment plan may depend on other factors such as the type, severity and cause of the crossbite⁴. You may want to consult a dentist or an orthodontist for a personalized assessment.  Source: Conversation with Bing, 8/24/2023  (1) Orthodontic treatment for posterior crossbites - Cochrane. https://bing.com/search?q=best+orthodontic+treatment+for+posterior+crossbites.  (2) Are braces effective for treating crossbite (top back teeth ... - Cochrane. https://www.cochrane.org/CD000979/ORAL_are-braces-effective-treating-crossbite-top-back-teeth-biting-down-inside-bottom-back-teeth.  (3) Orthodontic treatment for posterior crossbites - Cochrane. https://oralhealth.cochrane.org/news/orthodontic-treatment-posterior-crossbites.  (4) Orthodontic treatment for posterior crossbites - Ugolini, A - 2021 .... https://www.cochranelibrary.com/web/cochrane/content?templateType=information&urlTitle=%2Fcdsr%2Fdoi%2F10.1002%2F14651858.CD000979.pub3&doi=10.1002%2F14651858.CD000979.pub3&type=cdsr&contentLanguage=.  (5) Correction of Posterior Crossbites: Diagnosis and Treatment - AAPD. https://www.aapd.org/globalassets/media/publications/archives/binder-26-03.pdf. | Ugolini A, Agostino P, Silvestrini-Biavati A, Harrison JE, Batista KBSL. Orthodontic treatment for posterior crossbites. Cochrane Database of Systematic Reviews 2021, Issue 12. Art. No.: CD000979. DOI: 10.1002/14651858.CD000979.pub3.  Summary of evidence:  For children in the early mixed dentition stage (age 7 to 11 years old), quad‐helix and expansion plates are more beneficial than no treatment for correcting posterior crossbites. Expansion plates also increase the inter‐canine distance. Quad‐helix is more effective than expansion plates for correcting posterior crossbite and increasing inter‐molar distance. Treatment duration is shorter with quad‐helix than expansion plates.  For adolescents in permanent dentition (age 12 to 16 years old), Hyrax and Haas are similar for posterior crossbite correction and increasing the inter‐molar distance.  GRADE: 10/10 |
| 6. How do molar tubes compare to molar bands in terms of failure and decalcification? | Molar tubes and molar bands are two different methods used in orthodontic treatment to secure orthodontic appliances (e.g., braces) to the molars. Each method has its advantages and potential drawbacks when it comes to failure rates and the risk of decalcification (white spot lesions) on the teeth. Let's compare them:  **Molar Bands:**  1. **Advantages:**  - Bands are metal rings that encircle the entire molar tooth, providing strong and stable anchorage for orthodontic appliances.  - Bands are generally considered less likely to fail in terms of detachment from the tooth compared to tubes.  2. **Drawbacks:**  - Bands require more space on the tooth surface, which can be uncomfortable for the patient and may interfere with oral hygiene.  - Bands can sometimes contribute to decalcification because they create areas where plaque and food particles can accumulate more easily.  **Molar Tubes (Buccal Tubes or Bonded Molar Tubes):**  1. **Advantages:**  - Tubes are smaller and more discreet than bands, making them more comfortable for the patient.  - They are bonded (glued) to the tooth's surface, so they don't encircle the entire tooth, which can make oral hygiene easier.  - Tubes may be associated with a lower risk of decalcification compared to bands because they do not cover as much tooth surface.  2. **Drawbacks:**  - Tubes can have a slightly higher risk of detachment from the tooth compared to bands, particularly if not properly bonded or maintained.  - In some cases, tubes may require additional steps to remove adhesive residue from the tooth after treatment.  In summary, there is a trade-off between molar bands and molar tubes in terms of stability and the risk of decalcification:  - **Molar Bands** provide strong anchorage but may have a higher risk of decalcification due to their size and the difficulty of maintaining good oral hygiene around them.  - **Molar Tubes** are smaller and more comfortable, making them preferable for many patients, but they may have a slightly higher risk of detachment if not properly bonded.  Ultimately, the choice between molar bands and molar tubes should be made in consultation with your orthodontist, who will consider your specific orthodontic needs and the potential benefits and drawbacks of each option. Proper oral hygiene and maintenance, regardless of the method used, are essential to minimize the risk of decalcification and other oral health issues during orthodontic treatment. | Molar tubes and molar bands are both orthodontic appliances used to anchor other appliances or to attach archwires in orthodontic treatments. Here's a comparison between the two in terms of failure and decalcification:   1. **Failure**:    - **Molar Tubes**:      - These are directly bonded to the tooth surface.      - They can have a higher risk of failure in terms of detachment compared to molar bands, especially when they're exposed to strong masticatory forces or when they're not bonded correctly.      - The success of molar tubes also depends heavily on the quality of the bonding agent used, the tooth surface preparation, and the cleanliness of the tooth during the bonding process.    - **Molar Bands**:      - These are metal bands that encircle the molar entirely.      - They generally have a lower risk of detachment as they wrap around the tooth and are cemented in place. Their success also depends on the fit of the band and the quality of the cement used.      - They're traditionally used in cases where a higher load is expected, such as anchorage for space closure or when using headgear. 2. **Decalcification**:    - **Molar Tubes**:      - Since these are bonded directly to the tooth surface, there's a risk of decalcification around the periphery of the tube if proper oral hygiene is not maintained. However, the affected surface area tends to be smaller compared to bands.      - Bonding agents with fluoride-releasing properties can help reduce the risk of decalcification.    - **Molar Bands**:      - Decalcification can occur around the bands, especially if oral hygiene is poor and plaque accumulates. The risk area is typically larger due to the band covering more surface area on the tooth.      - Proper fitting of the band is essential to minimize spaces where plaque can accumulate.      - The advantage is that once the bands are removed, the part of the tooth covered by the band tends to be protected from decalcification. However, the edges where plaque can accumulate are at risk.   **Other considerations**:   - Some patients and clinicians might prefer molar tubes because they can be less bulky and more comfortable than bands. They also require fewer appointments since no prior band fitting is needed. - Molar bands can be more versatile as they can hold other attachments like hooks or tubes more securely. - In terms of aesthetics, molar tubes can be less noticeable, especially if they're made of ceramic or other tooth-colored materials.   In summary, both molar tubes and molar bands have their advantages and disadvantages in terms of failure and decalcification. The choice between them often depends on the specific orthodontic requirements, the patient's oral hygiene, and the orthodontist's preference. | Molar tubes and molar bands are both used to attach orthodontic brackets to molars. Molar tubes are bonded to the tooth, while molar bands are cemented to the tooth.  In terms of failure, molar bands are generally considered to be more reliable than molar tubes. This is because molar bands are more difficult to dislodge, as they are bonded to the tooth. Molar tubes, on the other hand, can be more easily dislodged, especially if they are not bonded properly.  In terms of decalcification, molar tubes have been shown to be associated with a higher risk of decalcification than molar bands. This is because the bonding process can damage the enamel, making it more susceptible to decalcification.  A 2013 study published in the journal *Evidence-Based Summaries in Dentistry* compared the failure and decalcification rates of molar tubes and molar bands. The study found that molar bands had a lower failure rate than molar tubes (25% vs. 30.5%). The study also found that molar bands were associated with a lower risk of decalcification than molar tubes (36% vs. 64%).  Another study, published in the journal *Orthodontics & Craniofacial Research* in 2019, found similar results. The study found that molar bands had a lower failure rate than molar tubes (28.8% vs. 30.5%) and were associated with a lower risk of decalcification (36% vs. 64%).  Based on the available evidence, it appears that molar bands are a more reliable and less decalcifying option than molar tubes. However, the best option for a particular patient will depend on the individual's circumstances. It is important to talk to your orthodontist to discuss the pros and cons of each option and decide which is best for you. | Based on the available evidence, **molar tubes have a higher failure rate and decalcification rate than molar bands**¹². One study found that the odds of bonding failure were approximately 3:1 in favor of molar bands². Another study found that the failure rate of molar tubes was 28.8% compared to 30.5% for molar bands after 12 months of follow-up³. However, the results may vary depending on the type of adhesive, cement and technique used for bonding or banding.  Source: Conversation with Bing, 8/24/2023  (1) Evaluation of failure rate of molar tubes with a modified bonding .... https://academic.oup.com/ejo/advance-article-abstract/doi/10.1093/ejo/cjad029/7226569.  (2) Bonded molar tubes associated with higher failure rate than molar bands. https://www.nature.com/articles/6400813.pdf.  (3) Molar tubes and failure rates A review - ResearchGate. https://www.researchgate.net/publication/371795569_Molar_tubes_and_failure_rates-A_review/fulltext/64959246c41fb852dd2736ea/Molar-tubes-and-failure-rates-A-review.pdf. | Millett DT, Mandall NA, Mattick RCR, Hickman J, Glenny AM. Adhesives for bonded molar tubes during fixed brace treatment. Cochrane Database of Systematic Reviews 2017, Issue 2. Art. No.: CD008236.  Summary of evidence:  The failure of molar tubes bonded with either a chemically‐cured or light‐cured adhesive was considerably higher than that of molar bands cemented with glass ionomer cement. One trial indicated that there was less decalcification with molar bands cemented with glass ionomer cement than with bonded molar tubes cemented with a light‐cured adhesive.  GRADE: 10/10 |
| 7. Do painkillers, taken before or after orthodontic treatment, help relieve pain? If so, which painkillers work best? | Painkillers can help relieve the discomfort and pain associated with orthodontic treatment, but it's important to use them as directed by your orthodontist or healthcare provider. The type and effectiveness of painkillers can vary from person to person, so it's a good idea to discuss your specific needs with your orthodontic provider. Here are some common pain relief options and considerations:  **1. Over-the-Counter Pain Medications:**  - **Nonsteroidal Anti-Inflammatory Drugs (NSAIDs):** NSAIDs such as ibuprofen (e.g., Advil, Motrin) are often recommended because they help reduce pain and inflammation. Taking an NSAID before your orthodontic appointment can help preemptively manage discomfort. Be sure to follow the recommended dosage and guidelines provided by your orthodontist or on the medication label.  - **Acetaminophen (e.g., Tylenol):** Acetaminophen is another option for pain relief. While it can help with pain, it is not as effective at reducing inflammation as NSAIDs. It is important not to exceed the recommended dose as excessive acetaminophen can be harmful to the liver.  **2. Topical Anesthetics:**  - Some orthodontic patients may experience discomfort or sore spots on the cheeks, lips, or tongue due to braces or other orthodontic appliances. Your orthodontist may recommend topical anesthetics (e.g., Orajel) to provide temporary relief from these localized irritations.  **3. Orthodontic Wax:**  - Orthodontic wax is not a painkiller, but it can be a useful tool to cover sharp or irritating edges of braces or orthodontic appliances. This can help prevent discomfort and irritation.  **4. Prescription Pain Medications:**  - In some cases, especially after more complex orthodontic procedures or oral surgeries, your orthodontist or oral surgeon may prescribe stronger pain medications to manage post-operative discomfort. It is crucial to follow the prescription and dosage instructions carefully.  **5. Ice Packs:**  - Applying ice packs to the outside of your mouth for short periods can help reduce swelling and provide some pain relief.  Remember that orthodontic discomfort and pain typically peak within the first few days after an adjustment or the placement of new orthodontic appliances. It then gradually subsides as your mouth adjusts to the changes. Consistently following your orthodontist's care instructions, maintaining good oral hygiene, and adhering to dietary restrictions (e.g., avoiding hard or sticky foods) can help minimize discomfort.  Always consult with your orthodontist or healthcare provider before taking any medication, especially if you have allergies or medical conditions that may interact with painkillers. They can provide personalized guidance on pain management during your orthodontic treatment. | Orthodontic treatment often causes discomfort or pain, particularly after adjustments are made to braces or after the placement of certain orthodontic appliances. The pressure exerted on the teeth to move them can result in soreness. Painkillers, both over-the-counter and prescription, can help manage this discomfort for many patients.   1. **Non-Steroidal Anti-Inflammatory Drugs (NSAIDs)**: These are some of the most commonly recommended painkillers for orthodontic pain.    - **Ibuprofen (e.g., Advil, Motrin)**: One of the most popular NSAIDs, it helps to reduce inflammation and relieve pain. It should be taken as directed and is best taken with food or milk to decrease the risk of stomach upset.    - **Naproxen (e.g., Aleve)**: Another NSAID, it has a longer duration of action compared to ibuprofen. As with ibuprofen, it's recommended to take it with food. 2. **Acetaminophen (e.g., Tylenol)**: This is not an NSAID but is effective at relieving pain. It does not have the anti-inflammatory properties of NSAIDs but is often well-tolerated and can be taken by those who cannot take NSAIDs due to allergies, gastrointestinal issues, or other contraindications. 3. **Aspirin**: This is an older pain reliever and has anti-inflammatory properties. It can help with pain, but it's generally not the first choice for children or teenagers due to the risk of Reye's syndrome. Always consult with a healthcare provider before giving aspirin to a child or teenager. 4. **Topical anesthetics**: Gels or ointments containing benzocaine can be applied directly to sore areas inside the mouth to numb them temporarily. These are often available over-the-counter.   **General Tips**:   - Taking painkillers before a scheduled orthodontic adjustment may help pre-emptively reduce pain. Some orthodontists recommend taking a painkiller an hour before the appointment to help manage discomfort. - Always follow the recommended dosage for over-the-counter painkillers and do not take more than the recommended amount. - If over-the-counter options do not relieve pain or if there are concerns about their use, consult with the orthodontist or a primary care provider. They may have additional recommendations or can prescribe stronger pain relievers if necessary. - In addition to painkillers, cold compresses applied externally to the face can help reduce inflammation and provide relief. Salt water rinses can also help soothe irritated oral tissues.   **Caution**: Always consult with a healthcare professional or pharmacist before starting any medication. They can provide guidance on appropriate dosages, potential drug interactions, and any other concerns related to the individual's health and the medications they are already taking. | There is no consensus on whether painkillers taken before or after orthodontic treatment are more effective in relieving pain. Some studies have shown that taking painkillers before treatment can help to reduce the pain, while others have shown that taking painkillers after treatment is more effective.  The best way to determine when to take painkillers is to talk to your orthodontist. They will be able to assess your individual situation and recommend the best course of treatment for you.  Here are some of the most common painkillers that are used to relieve pain during orthodontic treatment:   - Acetaminophen (Tylenol): Acetaminophen is a non-steroidal anti-inflammatory drug (NSAID) that is used to relieve pain and fever. It is generally considered to be safe for most people, but it can cause liver damage if taken in high doses or for a long period of time. - Ibuprofen (Advil, Motrin): Ibuprofen is also an NSAID that is used to relieve pain and fever. It is generally considered to be safe for most people, but it can cause stomach upset and bleeding in some people. - Naproxen (Aleve): Naproxen is another NSAID that is used to relieve pain and fever. It is generally considered to be safe for most people, but it can cause stomach upset and bleeding in some people. - Oral analgesics: Over-the-counter oral analgesics, such as Orajel® and Anbesol®, can help soothe sore teeth and gums.   If you are taking any other medications, be sure to talk to your doctor or pharmacist before taking painkillers. Some painkillers can interact with other medications, so it is important to be aware of the potential risks.  It is also important to follow the directions on the label when taking painkillers. Do not take more than the recommended dose, and do not take painkillers for more than the recommended period of time.  If you are experiencing severe pain, you should see your orthodontist. They may be able to adjust your braces or give you other pain relief measures. | Painkillers may help relieve pain caused by orthodontic treatment, but the effectiveness and safety of different painkillers may vary. A Cochrane review³ found that **ibuprofen** was more effective than placebo for reducing orthodontic pain, but there was not enough evidence to compare other painkillers or combinations of painkillers. However, some sources suggest that **acetaminophen** may be a better choice than ibuprofen or other nonsteroidal anti-inflammatory drugs (NSAIDs) for orthodontic pain, as NSAIDs may interfere with tooth movement and cause side effects such as stomach irritation or bleeding²⁴. You may want to consult your dentist or orthodontist for the best painkiller for your situation, and follow the dosage and instructions carefully.  Source: Conversation with Bing, 8/24/2023  (1) Painkillers for relieving pain caused by orthodontic treatment. https://www.cochrane.org/CD003976/ORAL_painkillers-relieving-pain-caused-orthodontic-treatment.  (2) Why Acetaminophen Is the Best Pain Medication When ... - Hodges Ortho. https://www.hodgesortho.com/blog/2020/11/17/why-acetaminophen-is-the-best-207609.  (3) The efficacy of analgesics in controlling orthodontic pain: a .... https://bmcoralhealth.biomedcentral.com/articles/10.1186/s12903-020-01245-w.  (4) Braces Pain: 10 Ways to Relieve Mouth Pain from Braces. https://childrensedationdentist.com/braces-pain-10-ways-relieve-mouth-pain-from-braces/.  (5) Oral Analgesics for Acute Dental Pain \| American Dental Association - ADA. https://www.ada.org/resources/research/science-and-research-institute/oral-health-topics/oral-analgesics-for-acute-dental-pain. | Monk AB, Harrison JE, Worthington HV, Teague A. Pharmacological interventions for pain relief during orthodontic treatment. Cochrane Database of Systematic Reviews 2017, Issue 11. Art. No.: CD003976. DOI: 10.1002/14651858.CD003976.pub2.  Summary of evidence:  There is moderate‐quality evidence that the use of analgesics reduces the pain associated with orthodontic treatment.  Due to a lack of evidence, we remain uncertain whether the systemic non steroidal anti‐inflammatories (NSAIDs) are more effective than paracetamol, and whether topical NSAIDs are more effective than local anaesthetic, at reducing pain associated with orthodontic treatment.  There is very low‐quality evidence that the use of pre‐emptive ibuprofen, taken one hour before orthodontic treatment, significantly reduces pain up to two hours after treatment; however, the effect appears to reduce over time, with no evidence of a difference at six hours and beyond.  GRADE: 10/10 |
| 8. Are removable better than fixed retainers? | The choice between removable and fixed retainers after orthodontic treatment depends on various factors, including the patient's individual needs, lifestyle, and orthodontic history. Both types of retainers have their advantages and disadvantages, and what's "better" may vary from person to person. Here's a comparison of removable and fixed retainers to help you understand their characteristics:  **Fixed Retainers:**  1. **Advantages:**  - **Continuous Retention:** Fixed retainers are bonded (glued) to the back of the teeth and provide continuous retention. They are always in place, which means they are effective 24/7 at maintaining the alignment of your teeth.  - **No Compliance Required:** Since fixed retainers are always on, there's no need to remember to wear them. They are a good option for patients who may struggle with compliance (wearing removable retainers as directed).  2. **Drawbacks:**  - **Oral Hygiene Challenges:** Fixed retainers can make it more challenging to clean between teeth, increasing the risk of plaque buildup and potential dental issues.  - **Maintenance:** Fixed retainers may require occasional maintenance or repair if they become loose or damaged.  **Removable Retainers:**  1. **Advantages:**  - **Better Oral Hygiene:** Removable retainers can be taken out for thorough oral hygiene, which may make it easier to maintain healthy gums and teeth.  - **No Dietary Restrictions:** You can remove removable retainers when eating, so there are no dietary restrictions.  2. **Drawbacks:**  - **Compliance Required:** The effectiveness of removable retainers depends on the patient's compliance. Patients must wear them as directed by their orthodontist, typically every night.  - **Risk of Loss or Damage:** Removable retainers can be lost or damaged if not properly cared for.  Ultimately, the choice between removable and fixed retainers should be made in consultation with your orthodontist, who will consider your specific needs and circumstances. In some cases, a combination of both types of retainers may be recommended.  For example, many orthodontists initially provide fixed retainers to ensure immediate post-treatment stability and then transition to removable retainers for long-term use. This combination can help address both the continuous retention needed in the early stages after treatment and the flexibility and ease of hygiene maintenance that removable retainers offer in the long run.  It's important to follow your orthodontist's instructions regarding retainer wear and maintenance to ensure the long-term success of your orthodontic treatment and maintain the beautiful smile you've achieved. | Both removable and fixed retainers have their advantages and disadvantages. The decision to choose one over the other often depends on individual preferences, the orthodontic history of the patient, and the specific recommendations of the orthodontist. Here's a comparison to help you better understand:  **Removable Retainers:**  **Advantages:**   1. **Easy to clean:** Patients can remove the retainer and brush and floss their teeth normally. 2. **Flexibility:** Patients can take them out for special events, photos, or during meals. 3. **Adjustability:** Orthodontists can make minor adjustments if needed. 4. **Visibility:** Some removable retainers are clear and almost invisible, such as Essix retainers.   **Disadvantages:**   1. **Losing or misplacing:** Because they can be taken out, they might be lost or forgotten. 2. **Less compliance:** Patients might forget to wear them the recommended amount, which can lead to teeth shifting. 3. **Less durability:** Depending on the type, some may wear out and need replacement more frequently. 4. **Potential for speech issues:** Some patients might experience speech difficulty initially.   **Fixed Retainers:**  **Advantages:**   1. **Constant support:** Since they're fixed, they provide 24/7 support to the teeth, preventing any relapse. 2. **No compliance issues:** Patients don't need to remember to put them on or take them off. 3. **Not easily lost:** They're bonded to the teeth, so there's no risk of misplacement. 4. **Minimal speech impact:** They are less likely to cause speech difficulties.   **Disadvantages:**   1. **Difficult cleaning:** Flossing especially can be more challenging with a fixed retainer. 2. **Potential for buildup:** If not cleaned properly, there's a risk for tartar or plaque accumulation. 3. **Can break or become unglued:** Though sturdy, they might break or the bonding can come off, requiring a dental visit. 4. **Long-term commitment:** They can remain on the teeth for many years, which some patients might not like.   In general, the decision will also be influenced by the orthodontic treatment you've had, the likelihood of your teeth moving back to their original position, and your personal preferences. It's important to discuss these factors with your orthodontist to make an informed decision. Regardless of the choice, the primary goal is to ensure that the teeth remain in their desired position after orthodontic treatment. | Removable retainers and fixed retainers are both used to keep teeth in their new position after orthodontic treatment. However, they have different advantages and disadvantages.  Removable retainers:   - Pros:   - They are easier to clean and maintain than fixed retainers.   - They can be removed for eating and brushing, which can make it easier to maintain good oral hygiene.   - They are less visible than fixed retainers. - Cons:   - They can be more easily lost or damaged.   - They may not be as effective as fixed retainers in preventing teeth from moving.   Fixed retainers:   - Pros:   - They are more secure than removable retainers and are less likely to be lost or damaged.   - They are more effective in preventing teeth from moving. - Cons:   - They can be more difficult to clean and maintain than removable retainers.   - They are more visible than removable retainers.   The best type of retainer for you will depend on your individual needs and preferences. If you are concerned about the visibility of your retainer, a removable retainer may be a better option for you. If you are concerned about the risk of losing or damaging your retainer, a fixed retainer may be a better option for you.  Ultimately, the decision of whether to use a removable or fixed retainer is up to you and your orthodontist. They will be able to assess your individual situation and recommend the best type of retainer for you.  Here are some additional things to consider when choosing between a removable and fixed retainer:   - Your age: Removable retainers are generally recommended for younger patients, as they are more likely to lose or damage fixed retainers. - Your lifestyle: If you are active or play sports, a fixed retainer may be a better option for you, as it is less likely to be dislodged. - Your oral hygiene: If you have difficulty cleaning your teeth, a removable retainer may be a better option for you, as it can be removed for cleaning. - Your budget: Fixed retainers are typically more expensive than removable retainers.   If you are concerned about the cost, visibility, or maintenance of different types of retainers, be sure to talk to your orthodontist. They can help you understand the different options and recommend the best type of retainer for you. | There is no definitive answer to this question, as both types of retainers have their pros and cons. **Fixed retainers** are permanently attached to the back of your teeth, so they provide constant stability and prevent relapse, but they may also cause plaque accumulation, tooth decay, or gingival inflammation if not cleaned properly¹⁴. **Removable retainers** can be taken out for eating and brushing, so they are easier to keep clean and may protect your teeth from grinding, but they may also be lost, damaged, or forgotten, and may not be as effective as fixed retainers for some cases¹³⁶. You may want to consult your orthodontist for the best type of retainer for your situation, and follow their instructions carefully.  Source: Conversation with Bing, 8/24/2023  (1) Fixed Retainer vs Removable Retainer: Which Is Right for You?. https://www.sportingsmiles.com/blog/fixed-retainer-vs-removable-retainer-which-is-right-for-you/.  (2) Permanent Retainers: Pros, Cons, Costs, and Vs. Removable - Healthline. https://www.healthline.com/health/permanent-retainer.  (3) Fixed vs Removable Retainers - Pan Orthodontics. https://www.panorthodontics.com/retainers-fixed-vs-removable/.  (4) Removable Retainer: Pros and Cons - Verywell Health. https://www.verywellhealth.com/removable-retainer-5217101.  (5) Comparison Between Removable and Fixed Orthodontic Retainers. https://www.neliti.com/publications/224514/comparison-between-removable-and-fixed-orthodontic-retainers.  (6) Fixed vs. Removable Retainers \| The Pros & Cons of Each. https://www.waltortho.com/fixed-vs-removable-retainers-the-pros-cons-of-each/. | Martin C, Littlewood SJ, Millett DT, Doubleday B, Bearn D, Worthington HV, Limones A. Retention procedures for stabilising tooth position after treatment with orthodontic braces. Cochrane Database Syst Rev. 2023 May 22;5(5):CD002283. doi: 10.1002/14651858.CD002283.pub5. PMID: 37219527; PMCID: PMC10202160.  Summary of evidence:  Teeth may return to their previous position more with part‐time wear of a removable retainer made of clear plastic than with a fixed retainer, but the difference was so small that it may not be important. If the removable retainer is worn full‐time, there may be no difference between the retainers in tooth movement. Clear plastic retainers worn part‐time may fail less than fixed retainers, but when worn full‐time may fail more. Better gum health may be achieved with clear plastic removable retainers than with fixed retainers, but no difference was found in tooth decay.  GRADE: 10/10 |
| 9. Does extracting wisdom teeth (third molars) affect crowding later in life? | The relationship between extracting wisdom teeth (third molars) and the potential for crowding later in life is a subject of debate among dental and orthodontic professionals. Here are some key points to consider:  1. **Wisdom Teeth and Crowding:** Wisdom teeth, also known as third molars, typically erupt in late adolescence or early adulthood. In many cases, there may not be enough space in the jaw to accommodate these additional teeth. As a result, they can become impacted (unable to fully erupt) or partially erupted, which can lead to various issues, including pain, infection, and damage to adjacent teeth.  2. **Extraction of Wisdom Teeth:** When wisdom teeth are causing problems or are at risk of causing problems in the future, dentists and oral surgeons often recommend their extraction. This is typically done to prevent or address issues like impaction, pain, infection, and damage to neighboring teeth.  3. **Crowding Concerns:** There is ongoing debate about whether the extraction of wisdom teeth can lead to crowding or shifting of the other teeth in the mouth later in life. Some studies have suggested a correlation between wisdom tooth extraction and a slight increase in the risk of crowding, while others have not found a significant link.  4. **Individual Variability:** The relationship between wisdom teeth and crowding appears to be highly individual. Factors like genetics, oral habits, and overall dental health may play a more significant role in the development of crowding than wisdom tooth extraction alone.  5. **Orthodontic Considerations:** If a patient is already prone to crowding or has undergone orthodontic treatment to address crowding issues, the orthodontist may take wisdom teeth into account when planning treatment. In some cases, wisdom tooth extraction may be recommended as a precautionary measure to prevent crowding relapse.  6. **Consultation with Dental Professionals:** If you have concerns about wisdom teeth and their potential impact on crowding, it's advisable to consult with your dentist or orthodontist. They can evaluate your specific situation, including your dental history, age, and overall oral health, to provide personalized recommendations.  In summary, while there is some debate about the impact of wisdom tooth extraction on crowding later in life, it is not a guaranteed cause of crowding. Many factors contribute to the development of dental crowding, and wisdom tooth extraction is just one of several potential factors. If you are considering wisdom tooth extraction or have concerns about crowding, it's best to discuss your specific situation with a qualified dental or orthodontic professional who can provide guidance based on your individual needs. | The relationship between wisdom teeth (third molars) and anterior dental crowding (crowding of the front teeth) has been the subject of much debate in dentistry for years.  Here's a summary of the research and prevailing thoughts as of my last update in 2021:   1. **Historical Belief**: Traditionally, it was believed by many dental professionals that wisdom teeth could exert pressure on the other teeth as they attempted to erupt, leading to crowding of the anterior teeth, especially the lower front teeth. Therefore, prophylactic (preventative) removal of third molars was sometimes recommended to prevent this crowding. 2. **Current Understanding**: More recent research has cast doubt on the direct causal relationship between wisdom teeth eruption and anterior crowding. Several studies have found that anterior crowding can occur whether or not wisdom teeth come in. Other factors, such as the natural forward drift of teeth that can happen with age, genetics, or late mandibular growth, might play a role in anterior crowding. 3. **Removal Reasons**: While anterior crowding might not be a solid standalone reason for the removal of wisdom teeth, there are other valid reasons for their removal:    - **Impaction**: This is when a tooth doesn't fully erupt because there isn't enough room in the mouth, or it's coming in at the wrong angle. This can lead to pain, swelling, and infection.    - **Cavities or Gum Disease**: Due to their location in the back of the mouth, wisdom teeth can be hard to clean, making them susceptible to cavities and gum disease.    - **Cyst Formation**: Rarely, a cyst can form around an impacted wisdom tooth, which can lead to bone destruction.    - **Orthodontic Reasons**: Sometimes, orthodontists may recommend removal of third molars to achieve better orthodontic outcomes or to ensure there's enough space for other teeth. 4. **Conservative Approach**: As our understanding evolves, there's a trend towards a more conservative approach. This means only removing wisdom teeth when there's a clear clinical need, rather than for purely prophylactic reasons.   It's important to remember that the decision to extract or retain wisdom teeth should be based on an individual's clinical needs. Regular dental check-ups are crucial so that your dentist can monitor the health and position of your wisdom teeth and advise you on the best course of action. If you or someone you know is considering wisdom teeth removal, it's essential to have a discussion with a dentist or oral surgeon to weigh the pros and cons specific to the individual's situation. | The answer to this question is not straightforward, as the effects of wisdom teeth extraction on crowding later in life can vary depending on the individual patient and the specific circumstances.  In general, wisdom teeth extraction is not thought to cause crowding later in life. However, there are some cases where it may be a contributing factor. For example, if wisdom teeth are impacted or partially erupted, they can cause crowding by pushing against the other teeth. In these cases, extracting the wisdom teeth may help to relieve the crowding and prevent it from getting worse.  It is also important to note that crowding can be caused by a number of other factors, such as genetics, the size of the teeth, and the size of the jaw. Therefore, even if wisdom teeth are extracted, crowding may still occur later in life if these other factors are also present.  Ultimately, the best way to determine whether wisdom teeth extraction will affect crowding later in life is to talk to your dentist or orthodontist. They will be able to assess your individual situation and recommend the best course of treatment for you.  Here are some additional things to consider when deciding whether or not to extract your wisdom teeth:   - The position of your wisdom teeth: If your wisdom teeth are impacted or partially erupted, they are more likely to cause crowding. - The size of your teeth: If your teeth are large, they are more likely to cause crowding, even if your wisdom teeth are not impacted. - The size of your jaw: If your jaw is small, there is less room for your teeth, which can lead to crowding. - Your overall health: If you have any medical conditions, such as diabetes or heart disease, you may be at increased risk of complications from wisdom teeth extraction. - Your wishes and preferences: Some people may prefer to have their wisdom teeth extracted to prevent any potential problems, while others may be willing to take the risk of crowding later in life.   If you are concerned about the potential effects of wisdom teeth extraction on crowding later in life, be sure to talk to your dentist or orthodontist. They can help you understand the risks and benefits of extraction and recommend the best course of treatment for you. | Hello, this is Bing. I searched the web for your question and found some information that may help you. According to some sources, there is **no scientific evidence** that wisdom teeth cause crowding of teeth¹², so extracting them will not prevent your teeth from becoming crooked. However, some other sources suggest that wisdom teeth can cause **damage to other teeth** or **infection** if they are impacted or don't have enough room to grow³⁴⁵⁶, so removing them may be necessary in some cases. You should consult your dentist for a professional opinion on whether you need wisdom teeth extraction or not. I hope this helps. 😊  Source: Conversation with Bing, 8/24/2023  (1) Do Wisdom Teeth Cause Crowding \| Seim Orthodontics. https://www.seimortho.com/do-wisdom-teeth-cause-crowding/.  (2) Wisdom teeth removal and crowding of teeth – the truth!. https://orthoatdonmills.com/wisdom-teeth-removal-and-crowding-of-teeth-the-truth/.  (3) Will My Teeth Shift After a Wisdom Tooth Extraction?. https://deandentalsolutions.com/will-my-teeth-shift-after-a-wisdom-tooth-extraction/.  (4) Wisdom tooth extraction - Mayo Clinic. https://www.mayoclinic.org/tests-procedures/wisdom-tooth-extraction/about/pac-20395268.  (5) Impacted wisdom teeth - Symptoms and causes - Mayo Clinic. https://www.mayoclinic.org/diseases-conditions/wisdom-teeth/symptoms-causes/syc-20373808.  (6) Wisdom teeth removal: When is it necessary? - Mayo Clinic. https://www.mayoclinic.org/diseases-conditions/wisdom-teeth/expert-answers/wisdom-teeth-removal/faq-20058558. | Turner S, Harrison JE, Sharif FN, Owens D, Millett DT. Orthodontic treatment for crowded teeth in children. Cochrane Database Syst Rev. 2021 Dec 31;12(12):CD003453. doi: 10.1002/14651858.CD003453.pub2. PMID: 34970995; PMCID: PMC8786262.  Summary of evidence:  Taking out wisdom teeth (third molars) does not seem to affect crowding later in life  GRADE: 10/10 |
| 10. Which wires work best for initial tooth alignment? | The choice of wires for initial tooth alignment in orthodontic treatment depends on several factors, including the patient's specific orthodontic needs, the treatment plan, and the orthodontist's preferences. Different types of orthodontic wires are used at various stages of treatment. In the initial stages, when tooth alignment is the primary focus, orthodontists typically use lighter and more flexible wires that allow for gradual tooth movement and alignment. Here are some common types of wires used for initial tooth alignment:  1. **Round Nickel-Titanium (Ni-Ti) Wires:** These wires are often used initially because they are flexible, easy to insert, and exert light forces on the teeth. They are well-suited for aligning and leveling teeth in the early stages of treatment. Ni-Ti wires can be used in both conventional braces and some aligner systems.  2. **Rectangular Nickel-Titanium Wires:** As the teeth start to align and level, orthodontists may progress to rectangular Ni-Ti wires. These wires provide more control over tooth movement and can help with torque and rotation corrections.  3. **Thermal Ni-Ti Wires:** These wires have properties that allow them to respond to the temperature of the mouth, becoming more flexible when warm and stiffer when cool. They can be effective for initial alignment as well as reducing the number of wire adjustments needed during treatment.  4. **Beta-Titanium Wires:** Beta-titanium wires offer a good balance between flexibility and force delivery. They are often used in the later stages of initial alignment and for more significant tooth movements.  5. **Stainless Steel Wires:** Stainless steel wires are generally used in the later stages of treatment when fine-tuning and detailing are required. They are stiffer and provide more control over tooth movement.  6. **Copper-Nickel-Titanium (Cu-Ni-Ti) Wires:** These wires combine the benefits of nickel-titanium and copper-nickel properties, offering both flexibility and the ability to apply gentle forces for tooth alignment.  The choice of which wire to use during initial tooth alignment will depend on the orthodontist's assessment of the patient's needs, the complexity of the case, and the desired treatment outcomes. Orthodontic treatment is highly individualized, and the orthodontist will design a treatment plan that may involve a progression of wire types as the teeth move into their desired positions.  It's essential to follow your orthodontist's instructions regarding wire changes and adjustments throughout the course of your treatment to achieve the best results safely and efficiently. | In orthodontics, wires (often referred to as archwires) are used to apply forces to teeth to move them to a desired position. The selection of an appropriate archwire depends on the specific requirements of the treatment phase and the desired biomechanical properties.  For initial tooth alignment, the following archwires are commonly used:   1. **Nitinol or Nickel-Titanium (NiTi) Wires**: These wires have superelastic properties, meaning they can be deformed but will return to their original shape, applying light, continuous forces on the teeth. This makes them ideal for initial tooth alignment because they can adapt to malaligned teeth and gently guide them into a better position. NiTi wires are very forgiving, accommodating a broad range of malocclusions without the need for frequent adjustments. 2. **Heat-activated NiTi Wires**: These are a variant of the NiTi wires. They are more flexible when cold and stiffen upon warming to body temperature. This can make them easier to insert into brackets, and once warmed in the mouth, they begin to exert their corrective force. 3. **Beta-titanium or TMA (Titanium Molybdenum Alloy) Wires**: They are sometimes used in the initial stages, especially in cases that might benefit from slightly stiffer properties than NiTi, but they're often more appropriate for later stages of treatment due to their stiffness and ability to maintain larger bends. 4. **Stainless Steel Wires**: These are usually reserved for the later stages of orthodontic treatment because they are stiffer and can deliver stronger forces. However, in some cases, thinner stainless steel wires might be used initially for specific needs.   The choice of wire is based on the patient's specific needs and the orthodontist's preference. Throughout orthodontic treatment, an orthodontist will typically progress through a series of wires with increasing stiffness and diameter to refine the position of the teeth and achieve the desired outcome.  As always, for specific clinical decisions, it's crucial to consult directly with an orthodontic specialist. | The best wires for initial tooth alignment are those that are strong enough to move the teeth but also flexible enough to avoid damaging the teeth or gums.  Some of the most common types of wires used for initial tooth alignment include:   - Nickel-titanium (NiTi) wires: NiTi wires are known for their superelasticity, which means they can return to their original shape after being deformed. This makes them ideal for initial tooth alignment, as they can be bent to a desired shape and then will slowly return to their original shape, moving the teeth in the desired direction. - Co-Cr wires: Co-Cr wires are made of a combination of cobalt and chromium, and are known for their strength and durability. They are not as elastic as NiTi wires, but they can be used to move teeth that are more resistant to movement. - Multistranded stainless steel wires: Multistranded stainless steel wires are made of multiple strands of stainless steel twisted together. They are strong and durable, but they are not as elastic as NiTi wires. They are often used as retainers after the initial tooth alignment has been completed.   The best type of wire for initial tooth alignment will depend on the individual patient's needs and the severity of their malocclusion. Your orthodontist will be able to assess your individual situation and recommend the best type of wire for you.  Here are some additional factors that your orthodontist may consider when choosing the best wire for initial tooth alignment:   - The severity of the malocclusion: The more severe the malocclusion, the stronger the wire that will be needed. - The patient's age: Younger patients tend to respond to orthodontic treatment more quickly than older patients, so they may be able to use a weaker wire. - The patient's overall health: Patients with certain medical conditions, such as diabetes or heart disease, may need to use a weaker wire. - The patient's wishes and preferences: Some patients may prefer to have a less visible wire, while others may be more concerned about the strength of the wire.   If you are concerned about the type of wire that will be used for your initial tooth alignment, be sure to talk to your orthodontist. They can help you understand the different options and recommend the best wire for you. | There is no definitive answer to this question, as different types of wires may have different advantages and disadvantages depending on the case. However, **nickel-titanium (NiTi) archwires** are commonly used for the initial leveling and alignment of the teeth due to their optimal elasticity, low stiffness, high flexibility, and high spring back¹³. There are also different variants of NiTi wires, such as **superelastic** and **copper-nickel-titanium (Cu-NiTi)**, that may offer better performance or comfort than conventional NiTi wires²³⁴. You may want to consult your orthodontist for the best choice of wires for your specific needs.  Source: Conversation with Bing, 8/24/2023  (1) Tooth alignment and pain experience with A-NiTi versus Cu-NiTi: a .... https://bing.com/search?q=best+wires+for+initial+tooth+alignment.  (2) Tooth alignment and pain experience with A-NiTi versus Cu-NiTi: a .... https://bmcoralhealth.biomedcentral.com/articles/10.1186/s12903-021-01789-5.  (3) Three-dimensional coupling between orthodontic bone remodeling and .... https://link.springer.com/article/10.1007/s00056-020-00259-x.  (4) A clinical comparison of three aligning archwires in terms of alignment .... https://meridian.allenpress.com/angle-orthodontist/article/85/3/434/98430/A-clinical-comparison-of-three-aligning-archwires.  (5) What are the best materials to use for the first arch wire ... - Cochrane. https://www.cochrane.org/CD007859/ORAL_what-are-best-materials-use-first-arch-wire-fixed-brace. | Wang Y, Liu C, Jian F, McIntyre GT, Millett DT, Hickman J, Lai W. Initial arch wires used in orthodontic treatment with fixed appliances. Cochrane Database Syst Rev. 2018 Jul 31;7(7):CD007859. doi: 10.1002/14651858.CD007859.pub4. PMID: 30064155; PMCID: PMC6513532.  Summary of evidence:  Moderate‐quality evidence shows that arch wires of coaxial superelastic nickel‐titanium (NiTi) can produce greater tooth movement over 12 weeks than arch wires made of single‐strand superelastic NiTi. Moderate‐quality evidence also suggests there may be no difference in pain at day 1 between multistrand stainless steel arch wires and superelastic NiTi arch wires. Other than these findings, there is insufficient evidence to determine whether any particular arch wire material is superior to any other in terms of alignment rate, time to alignment, pain and root resorption.  GRADE: 10/10 |

| **Questions** | **Evidence (literature)** |
| --- | --- |
| 1. Does orthodontic treatment affect the airway function? | Behrents RG, Shelgikar AV, Conley RS, Flores-Mir C, Hans M, Levine M, McNamara JA, Palomo JM, Pliska B, Stockstill JW, Wise J, Murphy S, Nagel NJ, Hittner J. Obstructive sleep apnea and orthodontics: An American Association of Orthodontists White Paper. Am J Orthod Dentofacial Orthop. 2019 Jul;156(1):13-28.e1. doi: 10.1016/j.ajodo.2019.04.009. PMID: 31256826. |
| 2. Is early orthodontic treatment in two phases for children with prominent upper teeth, more beneficial compared to treatment that is provided in one phase in adolescence? | Batista KBSL, Thiruvenkatachari B, Harrison JE, O'Brien KD. Orthodontic treatment for prominent upper front teeth (Class II malocclusion) in children and adolescents. Cochrane Database of Systematic Reviews 2018, Issue 3. Art. No.: CD003452. |
| 3. Do light vibrational forces reduce the duration of orthodontic treatment? | El-Angbawi A, McIntyre G, Fleming PS, Bearn D. Non-surgical adjunctive interventions for accelerating tooth movement in patients undergoing orthodontic treatment. Cochrane Database Syst Rev. 2023 Jun 20;6(6):CD010887. doi: 10.1002/14651858.CD010887.pub3. PMID: 37339352; PMCID: PMC10281004. |
| 4. How long should one wait to go for surgical exposure after an interceptive intervention for palatally displaced permanent canine? | Benson PE, Atwal A, Bazargani F, Parkin N, Thind B. Interventions for promoting the eruption of palatally displaced permanent canine teeth, without the need for surgical exposure, in children aged 9 to 14 years. Cochrane Database Syst Rev. 2021 Dec 30;12(12):CD012851. doi: 10.1002/14651858.CD012851.pub2. PMID: 34967448; PMCID: PMC8717471. |
| 5. What is the best orthodontic treatment for posterior crossbites? | Ugolini A, Agostino P, Silvestrini-Biavati A, Harrison JE, Batista KBSL. Orthodontic treatment for posterior crossbites. Cochrane Database of Systematic Reviews 2021, Issue 12. Art. No.: CD000979. DOI: 10.1002/14651858.CD000979.pub3. |
| 6. How do molar tubes compare to molar bands in terms of failure and decalcification? | Millett DT, Mandall NA, Mattick RCR, Hickman J, Glenny AM. Adhesives for bonded molar tubes during fixed brace treatment. Cochrane Database of Systematic Reviews 2017, Issue 2. Art. No.: CD008236. |
| 7. Do painkillers, taken before or after orthodontic treatment, help relieve pain? If so, which painkillers work best? | Monk AB, Harrison JE, Worthington HV, Teague A. Pharmacological interventions for pain relief during orthodontic treatment. Cochrane Database of Systematic Reviews 2017, Issue 11. Art. No.: CD003976. DOI: 10.1002/14651858.CD003976.pub2. |
| 8. Are removable better than fixed retainers? | Martin C, Littlewood SJ, Millett DT, Doubleday B, Bearn D, Worthington HV, Limones A. Retention procedures for stabilising tooth position after treatment with orthodontic braces. Cochrane Database Syst Rev. 2023 May 22;5(5):CD002283. doi: 10.1002/14651858.CD002283.pub5. PMID: 37219527; PMCID: PMC10202160. |
| 9. Does extracting wisdom teeth (third molars) affect crowding later in life? | Turner S, Harrison JE, Sharif FN, Owens D, Millett DT. Orthodontic treatment for crowded teeth in children. Cochrane Database Syst Rev. 2021 Dec 31;12(12):CD003453. doi: 10.1002/14651858.CD003453.pub2. PMID: 34970995; PMCID: PMC8786262. |
| 10. Which wires work best for initial tooth alignment? | Wang Y, Liu C, Jian F, McIntyre GT, Millett DT, Hickman J, Lai W. Initial arch wires used in orthodontic treatment with fixed appliances. Cochrane Database Syst Rev. 2018 Jul 31;7(7):CD007859. doi: 10.1002/14651858.CD007859.pub4. PMID: 30064155; PMCID: PMC6513532. |
