# Supplementary material for: Evidence-based potential of generative artificial intelligence large language models in orthodontics: a comparative study of ChatGPT, Google Bard, and Microsoft Bing
Source: Eur J Orthod. 2024 Apr 13;48(1):cjae017. doi: 10.1093/ejo/cjae017 (PMC12810200; doi:10.1093/ejo/cjae017)
Supplement: cjae017_suppl_Supplementary_Table_2 [file cjae017_suppl_supplementary_table_2.docx]

**Supplementary Table 2.**

**ASSESSMENT RUBRIC**

| **Criteria** | **0** | **1-2** | **3-4** | **5-7** | **8-9** | **10** | **Rating** |
| --- | --- | --- | --- | --- | --- | --- | --- |
| **Comprehensiveness**  **[25%]** | Response is not at all comprehensive | Very few key points are included | A basic level of key points is included | Many key points are included, but still some key information is missing | Almost all key points are included | All key points are included |  |
| **Scientific accuracy**  **[25%]** | Response completely inaccurate | Response mostly inaccurate | Response presents a basic level of accuracy | Response exhibits a good level of scientific accuracy, but some key points are not presented accurately | Almost all key points are presented accurately | All key points are presented accurately |  |
| **Clarity**  **[25%]** | Response not clear at all, leaving the assessor very confused | Response obscures most key points | Response obscures many key points | Response is mostly clear, but some key points are not | Almost all key points are presented clearly | All key points are presented clearly |  |
| **Relevance**  **[25%]** | Response not relevant at all | Many irrelevant points included | Some irrelevant points included | Most points included are relevant | Almost all points included are relevant | All points included are relevant |  |
| **Total score [rounded to the next integer]** | | | | | | |  |
